# G‐type Halohydrin Dehalogenases Catalyze Ring Opening Reactions of Cyclic Epoxides with Diverse Anionic Nucleophiles**

**DOI:** 10.1002/chem.202202343

**Published:** 2022-11-14

**Authors:** Jennifer Solarczek, Felix Kaspar, Pia Bauer, Anett Schallmey

**Affiliations:** ^1^ Technische Universität Braunschweig Institute for Biochemistry Biotechnology and Bioinformatics Spielmannstraße 7 38106 Braunschweig Germany; ^2^ Chair of Bioprocess Engineering Technische Universität Berlin Ackerstraße 76 13355 Berlin Germany; ^3^ Amedes Genetics MVZ for Laboratory Medicine Georgstraße 50 30159 Hannover Germany; ^4^ Zentrum für Pharmaverfahrenstechnik (PVZ) Technische Universität Braunschweig Franz-Liszt-Str. 35a 38106 Braunschweig Germany; ^5^ Braunschweig Integrated Center of Systems Biology (BRICS) Technische Universität Braunschweig Rebenring 56 38106 Braunschweig Germany

**Keywords:** β-substituted alcohols, biocatalysis, lyases, stereoselectivity, thermostability

## Abstract

Halohydrin dehalogenases are promiscuous biocatalysts, which enable asymmetric ring opening reactions of epoxides with various anionic nucleophiles. However, despite the increasing interest in such asymmetric transformations, the substrate scope of G‐type halohydrin dehalogenases toward cyclic epoxides has remained largely unexplored, even though this subfamily is the only one known to display activity with these sterically demanding substrates. Herein, we report on the exploration of the substrate scope of the two G‐type halohydrin dehalogenases HheG and HheG2 and a newly identified, more thermostable member of the family, HheG3, with a variety of sterically demanding cyclic epoxides and anionic nucleophiles. This work shows that, in addition to azide and cyanide, these enzymes facilitate ring‐opening reactions with cyanate, thiocyanate, formate, and nitrite, significantly expanding the known repertoire of accessible transformations.

## Graphical abstract

**G‐type halohydrin** dehalogenases convert 5‐ and 6‐membered cyclic epoxides with a range of small anionic C‐, N‐, O‐ and S‐nucleophiles.

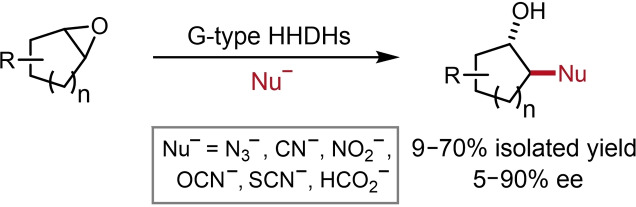

## Introduction

**Scheme 1:**
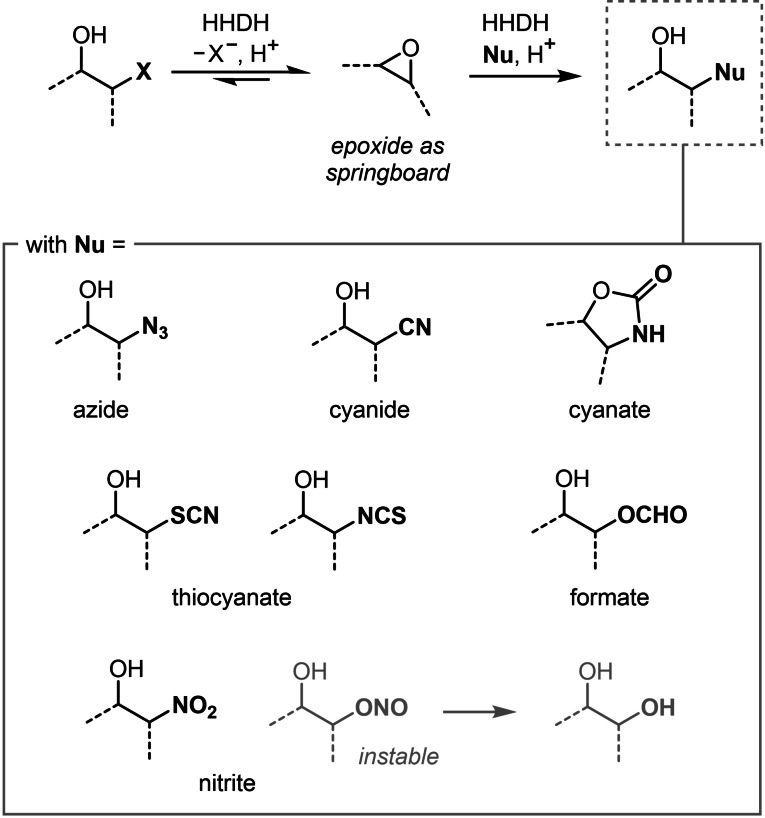
Halohydrin dehalogenase‐catalyzed epoxide ring opening with a range of C‐, N‐, O‐, S‐ and halide (X=Cl, Br) nucleophiles.

Halohydrin dehalogenases (HHDHs; also referred to as haloalcohol dehalogenases) are privileged enzymes, as they exhibit a unique ability to catalyze substitution reactions with a variety of (in‐)organic nucleophiles.^[1]^ Natively, HHDHs perform the dehalogenation of *β*‐haloalcohols via formation of the corresponding epoxide.^[2]^ Their ability to also catalyze the reverse reaction (i. e. epoxide ring opening) with anionic C‐, N‐, O‐, S‐ and halide nucleophiles^[3]^ provides divergent access to an impressive repertoire of valuable products[^4^, ^5^, ^6^, ^7^, ^8^, ^9^] (Scheme 1). This catalytic promiscuity, combined with the diversity of sequences available from databases, has made HHDHs attractive biocatalysts for kinetic resolutions and desymmetrization reactions.[^10^, ^11^, ^12^] For instance, HHDHs have been employed for the synthesis of enantioenriched oxazolidinones, tertiary alcohols, epihalohydrins, and – more recently – cyclohexyl synthons, spiro‐epoxyoxindoles and benzylic alcohols.[^10^, ^13^, ^14^, ^15^, ^16^, ^17^, ^18^, ^19^]

Despite the breadth of literature examples available for the application of HHDHs in asymmetric catalysis, these enzymes come with some limitations, which have thus far impeded their wider application in (chemo‐)enzymatic synthesis.^[12]^ Chief among these is their relatively specific substrate scope, which typically only includes terminal epoxides.^[20]^ As such, the scarcity of HHDHs for the conversion of sterically demanding internal epoxides limits the chemical space accessible with these enzymes.[^21^, ^22^] Since the catalytic promiscuity of HHDHs varies immensely between their different subtypes,[^22^, ^23^] the identification of new, catalytically promiscuous HHDHs is of current interest for the development of new asymmetric biocatalytic processes.

Motivated by this challenge, we sought to identify HHDHs with improved catalytic properties, including a broader substrate scope and, ideally, a high (thermal) stability under operational conditions. Recently, we reported on the characterization of thermostabilized mutants of HheG from *Ilumatobacter coccineus*.^[24]^ Unlike most other HHDHs, wild‐type HheG exhibits ring‐opening activity with cyclic epoxides such as cyclohexene oxide (**1**) and limonene oxide (**2**).^[22]^ However, HheG is comparably thermolabile ($T_{m}$ =38 °C in Tris‐EDTA buffer containing 10 % (v/v) glycerol)^[24]^ and shows only moderate enantioselectivity, for example in the desymmetrization of cyclohexene oxide (ca. 45 % ee).[^22^, ^24^] We found that mutations in a distal loop region confer increases in stability and activity, presumably by regulating loop dynamics.^[24]^ Moreover, as HheG is remarkably easy to crystallize, its immobilization as cross‐linked enzyme crystals yields considerable increases in stability, too, even under various reaction conditions.^[25]^ In addition, a close relative of HheG, HheG2 from *I. nonamiensis* (74 % sequence identity to HheG), also exhibited significant activity with the studied cyclic epoxides **1** and **2**.^[22]^ Based on these findings, we were intrigued by the catalytic potential of the G‐type HHDHs and hypothesized that these enzymes could provide synthetically useful activities with other cyclic epoxides. As HheG and HheG2 were previously only examined with azide and cyanide as nucleophiles,^[22]^ but the acceptance of other anionic nucleophilic species such as formate, cyanate, thiocyanate, and nitrite is known for HheC,^[3]^ we envisioned that a diversification of the nucleophile scope in the G‐type HHDHs could open new opportunities for biocatalytic reaction engineering. Herein, we report on the exploration of the substrate scope of HheG, HheG2 and a newly identified, more thermostable member of the family, HheG3, with a variety of sterically demanding cyclic epoxides and anionic nucleophiles. This analysis extends the known catalytic repertoire of HHDHs and reveals thermostability under operational conditions as a major determinant for HHDH productivity.

## Results and Discussion

**Figure 1:**
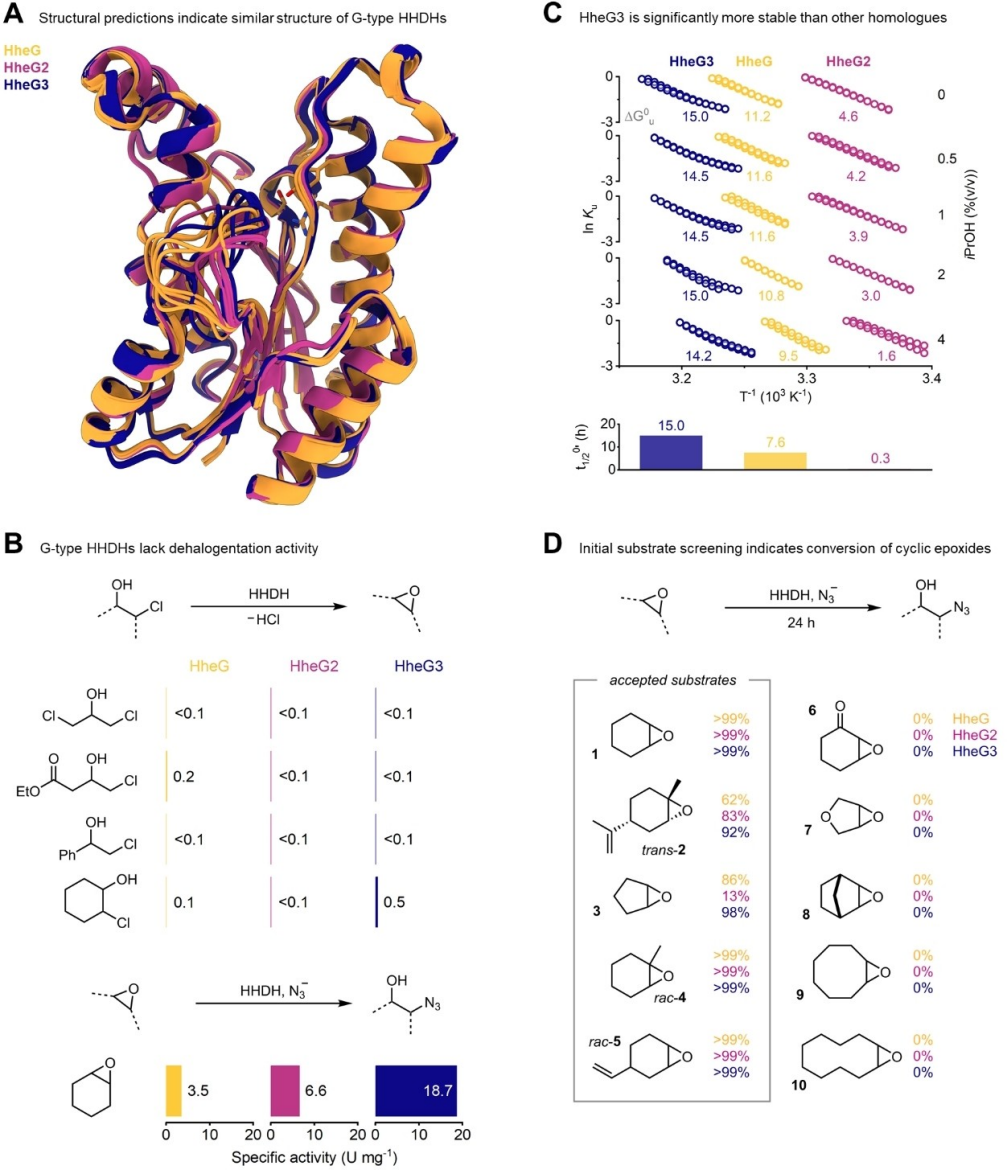
Characterization of G‐type HHDHs. A) Superposition of the five best predicted models^[29]^ for HheG, HheG2 and HheG3 and the experimental crystal structure of HheG (PDB ID 5o30^[22]^) indicates high structural similarity, expect for the flexible 15 amino acid loop region unique to the G‐type HHDHs. The catalytic triad is shown as sticks and reflects the residue positions in the experimental structure of HheG. B) G‐type HHDHs largely lack dehalogenation activity, as determined by a halide release assay, but are highly active in the nucleophilic ring opening of cyclohexene oxide, setting them apart from other known HHDHs. C) Arrhenius plots obtained by differential scanning fluorimetry reveal that HheG3 is much more stable and cosolvent‐resistant than its relatives, which translates to a longer apparent half‐life under typical operational conditions. D) The catalytic promiscuity with azide as the nucleophile enables the three G‐type HHDHs to convert a series of other cyclic epoxides. Please see the Supporting Information for experimental details.

We began our studies of the G‐type HHDHs with a re‐analysis of the available sequence space. HHDHs belong to the superfamily of short‐chain dehydrogenases/reductases[^26^, ^27^] and are distinguished by characteristic sequence motifs, including a Ser‐Tyr‐Arg catalytic triad.^[28]^ Since the identification of HheG and HheG2 by database mining in 2014 and 2017,[^22^, ^23^, ^28^] respectively, advances in sequencing technology have expanded the available sequence space considerably. Accordingly, mining this expanded sequence space for putative HHDH sequences based on known sequence motifs^[28]^ and classification of the hits into phylogenetic subtypes yielded a new member of the G subtype, HheG3 from *Actinobacteria bacterium*. This enzyme exhibits 70 % sequence identity with HheG and alignment of the protein sequences indicates a high degree of conservation around the catalytic triad and first‐sphere residues (Figure S1). Structural predictions by AlphaFold2[^29^, ^30^] suggest a very similar tertiary structure of HheG3 compared to HheG and HheG2 (except for the flexible loop unique to the G‐type HHDHs), all of which agree well with the experimental crystal structure of HheG^[22]^ (Figure 1A). In analogy to its relatives,[^22^, ^23^] HheG3 possesses almost no dehalogenation activity with a variety of *β*‐chloroalcohols but proved highly active in the nucleophilic ring opening of the cyclic epoxide **1** with azide (Figure 1B), confirming HheG3’s classification as a G‐type HHDH.

Next, we assessed the thermostability of HheG, HheG2 and HheG3 under operational conditions, which typically require the application of organic cosolvents to accommodate hydrophobic epoxide substrates. Since even moderate concentrations of organic cosolvents are known to affect the stability and activity of HHDHs,[^24^, ^31^] we opted to use *i*PrOH, which was tolerated best by HheG in previous studies.^[24]^ Differential scanning fluorimetry of the three enzymes incubated in Tris buffer at pH 7 with 3 % (v/v) glycerol revealed that different concentrations of *i*PrOH drastically lowered the apparent melting points ($T_{m}$ ) of all three enzymes (Figure 1C). Despite its favorable catalytic performance in previous studies,^[22]^ HheG2 displayed $T_{m}$ values of only 29–25 °C, which translated to apparent Gibbs free energies of unfolding ($\Delta G_{u}^{0}$ ) at 25 °C of 4.6 (with 0 % *i*PrOH) to 1.6 kJ mol^−1^ (with 4 % *i*PrOH). In contrast, HheG and HheG3 exhibited much higher $T_{m}$ and $\Delta G_{u}^{0}$ values (Figure 1C), with HheG3 almost performing on par with the thermostabilized variants T123G and T123W of HheG reported recently.^[24]^ Curiously, HheG3 possesses a methionine at the position corresponding to the critical T123 in HheG. Since the variant T123M was either not present in our previous library screen for the T123 position in HheG^[24]^ or did not exhibit an increased thermostability, the origins of HheG3’s increased stability currently remain elusive. In accordance with their $T_{m}$ values, HheG and HheG3 displayed much higher stability under typical reaction conditions, as both enzymes retained measurable activity in the azidolysis of **1** after 24 h of incubation, while HheG2 was rapidly inactivated (Figure 1C).

**Figure 2:**
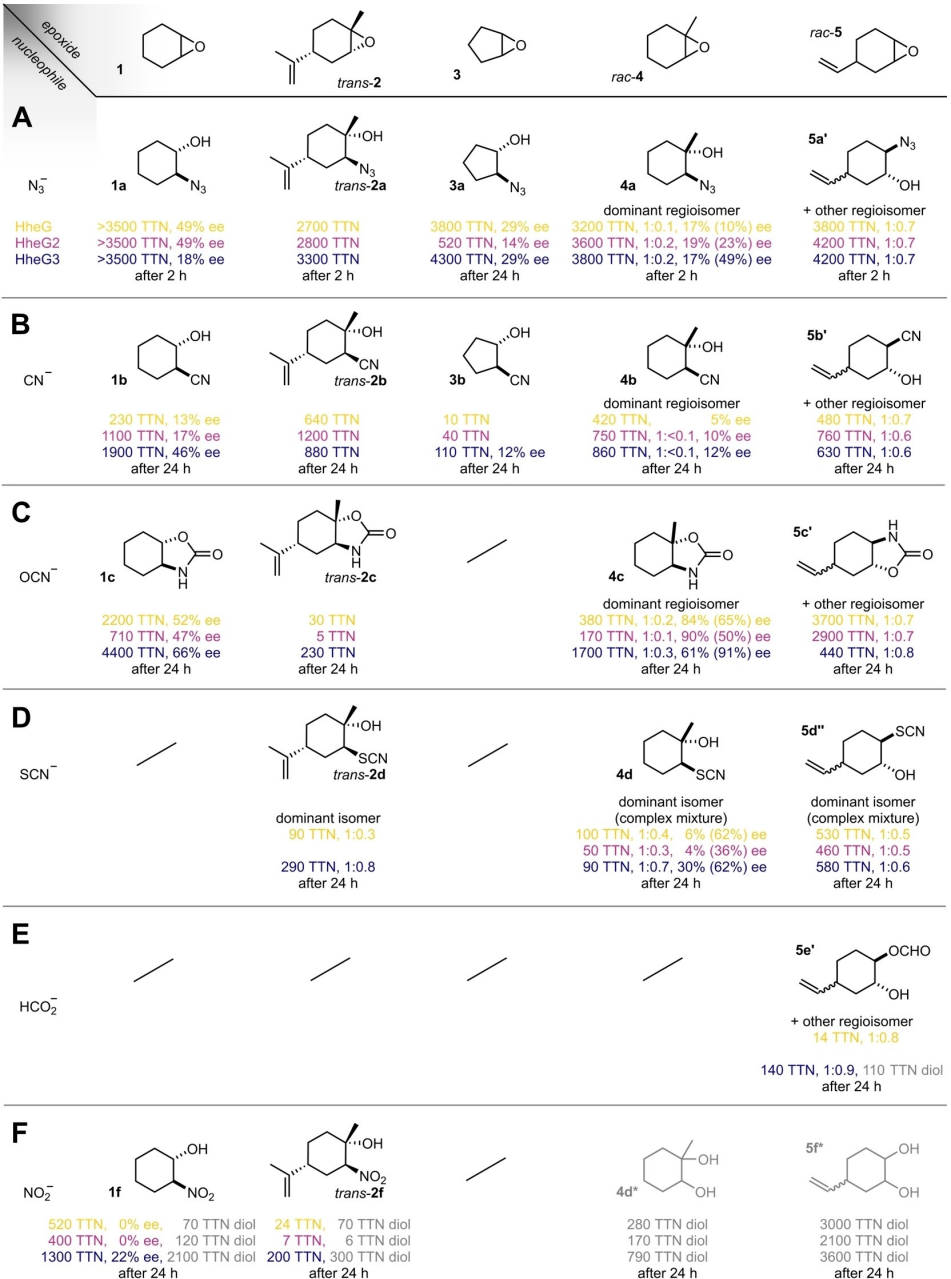
Substrate scope of the G‐type HHDHs. Bold bonds indicate relative configurations and wedges represent absolute configurations. Typical reaction conditions were 20 mM epoxide, 40 mM nucleophile, and 200 μg HHDH in 1 mL of 50 mM Tris⋅SO_4_ buffer with 2 % *i*PrOH at pH 7 and 22 °C. TTNs were determined by analysis of crude reaction mixtures by achiral GC and refer to the sum of all formed product isomers. The ratios refer to the ratio of formed regioisomers (e. g. **4 a** vs. **4 a′**), except for reactions with thiocyanate, where given ratios refer to the ratio of isomers arising from *S : N*‐nucleophilic attack. These values are normalized to the isomer shown. Enantiomeric excesses were determined by analysis of the crude reaction mixtures by chiral GC and also refer to the isomer shown. The ee values in parentheses refer to the corresponding other (regio)isomer. Please see the Supporting Information for the full experimental details and analytical procedures.

**Figure 3:**
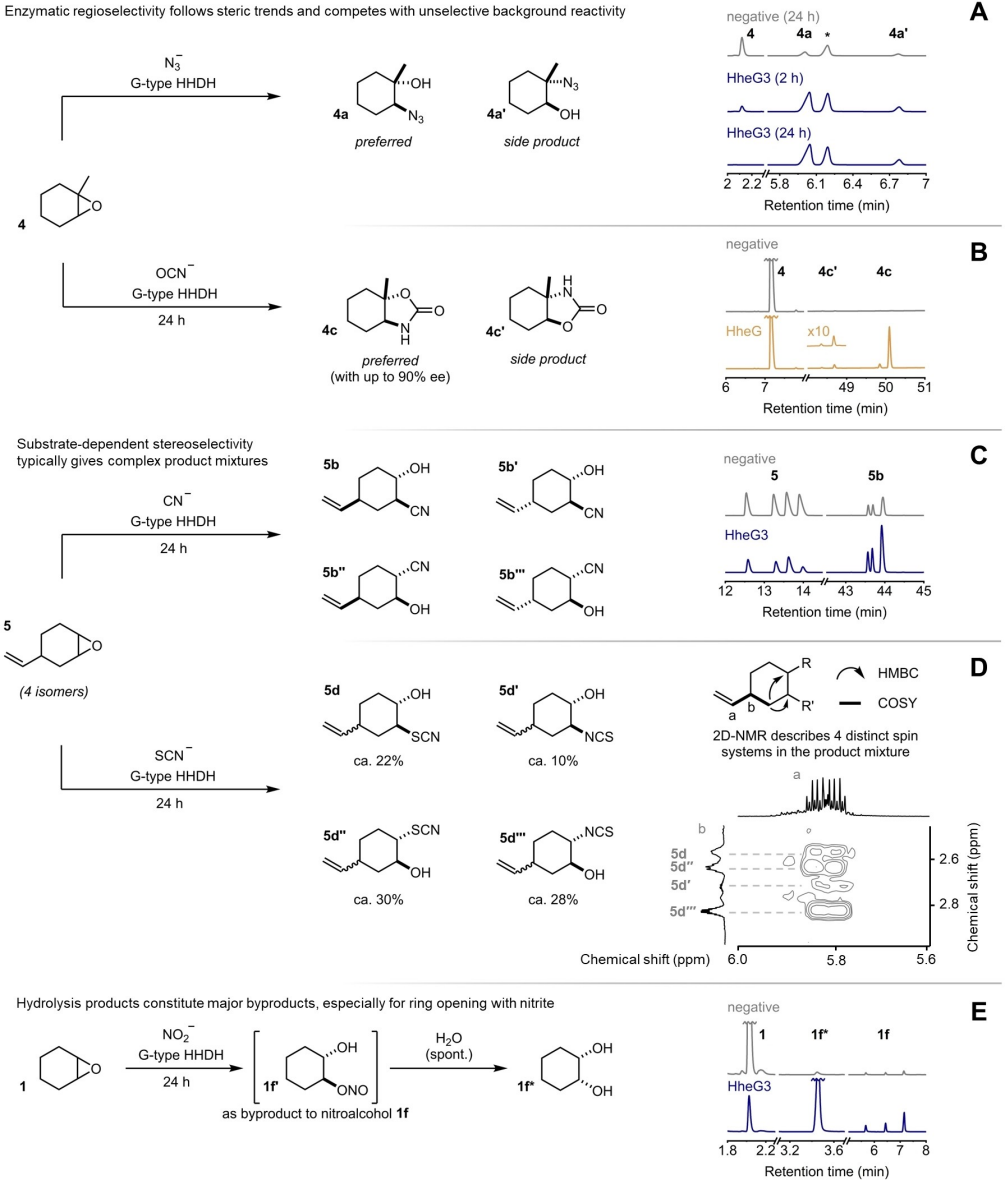
Illustrative analytical data for selected HHDH‐catalyzed ring‐opening reactions. *internal standard.

To further probe the scope of accepted cyclic substrates, we initially challenged the three G‐type HHDHs with the model substrates **3**–**10** using azide as the nucleophile (Figure 1D). In addition to the known substrates **1** and **2**, all enzymes displayed activity with cyclopentene oxide (**3**), the α‐methylated epoxide **4** and a cyclic epoxide bearing a distal vinyl group (**5**) with most reactions reaching nearly full conversion after 24 h. In contrast, electronically challenging substrates such as the ketone **6** or tetrahydrofurane **7** were not converted. Similarly, incubation of the highly sterically demanding bridged tricycle **8** and the octa‐ and decacycles **9** and **10** with the three enzymes gave no conversion to the corresponding azidoalcohols. Following these initial tests with purified enzyme, we additionally conducted preparative‐scale reactions with **3**–**5**, azide and whole cells harboring HheG, which yielded azidoalcohols **3 a**–**5 a** in moderate to good yield. Analysis by chiral GC and NMR revealed that all products were obtained as mixtures of regioisomers with low to moderate enantiomeric excess but generally high total turnover numbers (TTNs, Figure 2A). For instance, the azidolysis of **4** with 0.02 mol% HheG3 proceeds with full consumption of the starting material and a significant preference for attack at the sterically less hindered site to give azidoalcohol **4 a**. In addition, the corresponding regioisomer **4 a’** after nucleophilic attack at the sterically more hindered site (Figure 3A) is formed in lower amounts but with higher enantiomeric excess. Overall, product profiles did not change when switching from small‐scale reactions using purified enzymes to whole‐cell reactions on preparative scale, which is in agreement with previous work on HHDH‐catalyzed epoxide ring‐opening reactions.^[22]^

Motivated by these initial successes, we proceeded to examine the promiscuity of HheG, HheG2 and HheG3 in the ring‐opening reaction of **1**–**5** with other nucleophiles. To this end, we tested all combinations of **1**–**5** with the nucleophiles cyanide, cyanate, thiocyanate, formate, and nitrite with purified G‐type HHDHs under catalyst‐limiting conditions. Reactions showing the formation of ring‐opened product were scaled up with the best‐performing HHDH in preparative whole‐cell biotransformations to verify the formation of the anticipated product by NMR and HRMS. These standards were then used to assign peaks obtained by analysis of crude reaction mixtures by achiral GC to calculate TTNs of all enzymes with the tested epoxide‐nucleophile combinations. In total, 15 of the 25 possible ring‐opened products could be obtained (Figure 2), although TTN and selectivity varied greatly between different epoxide‐nucleophile combinations and different enzymes. For instance, ring‐opening of **1**–**5** with cyanide by HheG3 proceeded with synthetically useful TTNs and generally provided low to moderate enantioenrichment in the products. While the α‐methylated cyanoalcohol **4 b** was obtained almost as a single regioisomer (Figure 2B), all four isomers of the racemic epoxide **5** were converted, affording a complex and inseparable mixture of product isomers (Figure 3C). A similar situation existed with the combination of thiocyanate and **5**. Here, in addition to the formation of all possible regio‐ and stereoisomers, NMR analysis of the product mixture confirmed the formation of the isomers arising from *S*‐ and *N*‐nucleophilic attack of thiocyanate at the epoxide (Figure 3D), with a preference for the *S*‐nucleophilic attack (Figure 2D). This is in contrast to a previous report on HheC for the ring opening of 1,2‐epoxybutane with thiocyanate, which yielded the corresponding thioether product exclusively.^[3]^ Respective product isomers resulting from *S*‐ and *N*‐nucleophilic attack of thiocyanate at the sterically less hindered carbon atom were also obtained with epoxides **2** and **4**, in the latter case even with moderate enantioenrichment (Figure 2D). In contrast, HHDH‐catalyzed ring‐opening with cyanate generally proved much more selective and provided moderate to high enantiocontrol for the oxazolidinones arising from **1** and **4**, as HheG and HheG2 afforded **4 c** with >80 % ee (Figure 3B). This result is in good agreement with previous reports from the Chen group who showed that HheG confers moderate enantiocontrol in the ring opening of styrene oxides with cyanate.[^13^, ^19^] With formate as the nucleophile, only **5** gave any ring‐opened product yielding the corresponding formate ester, which partially hydrolyzed to the respective diol as well.

While the *trans*‐diols arising from non‐enzymatic hydrolysis of the epoxides in the aqueous reaction mixture constituted minor byproducts in all cases, ring‐opening with nitrite additionally yields the *cis*‐diols as possible products via hydrolysis of the corresponding nitrite esters. The latter are formed upon *O*‐nucleophilic attack (Figure 3E), while *N*‐nucleophilic attack at the epoxide results in formation of stable nitroalcohols.^[32]^ The nitroalcohols **1 f** and **2 f** could be obtained with all three enzymes with low to moderate TTNs, whereas the nitroalcohols arising from **3**–**5** were not observed. Instead, significant amounts of diol were obtained in reactions with epoxides **4** and **5**. Hence, the chemoselectivity of all three enzymes for formation of either nitroalcohol or nitrite ester in epoxide ring opening reactions with nitrite seems to be substrate‐dependent.

Collectively, this analysis revealed that the G‐type HHDHs harbor significant promiscuity regarding the accepted epoxides and nucleophiles. In addition to slight activity differences of the three HHDHs (Figure 1B), the catalytic performance of these enzymes appeared to be largely determined by their stability under operational conditions. As a general trend, the labile HheG2 gave the lowest TTNs and converted the fewest epoxides, while the much more stable HheG3 proved the most productive of the three enzymes. However, our dataset presents several exceptions to this trend (Figure 2, for example **2 b**, **5 b** and **5 c**) and studies to elucidate the reasons for this atypical behavior are ongoing.

## Conclusion

In conclusion, we have identified and characterized a new member of the G subtype of HHDHs and explored the substrate promiscuity of this enzyme and its relatives regarding their ring‐opening activity with cyclic epoxides and a range of anionic nucleophiles. In contrast to other known HHDHs, G‐type HHDHs convert 5‐ and 6‐membered cyclic epoxides with a range of small anionic C‐, N‐, O‐ and S‐nucleophiles. Although the selectivity and activity of these enzymes toward several of the investigated epoxide‐nucleophile combinations would need to be improved to deliver high yields of enantioenriched product, we anticipate that this can be achieved through protein engineering supported by computational design.^[33]^ As such, the catalytic promiscuity displayed by the G‐type HHDHs outlined herein presents an ideal starting point for such campaigns. Lastly, we believe that the additional stability and cosolvent resistance of the newly identified HheG3 will prove useful in this regard, as the introduction of thermal and solvent stability in G‐type HHDHs by means of protein engineering has historically been challenging.^[24]^

## Experimental Section

**Protein expression and purification**: Heterologous enzyme production of halohydrin dehalogenases HheG, HheG2 as well as HheG3 was carried out as reported previously.[^22^, ^24^] Briefly, a preculture of the respective *E. coli* expression strain was grown in LB medium (10 g L^−1^ tryptone, 5 g L^‐1^ yeast extract, 10 g L^−1^ NaCl) overnight, which was used to inoculate an expression culture in TB medium (12 g L^−1^ tryptone, 24 g L^−1^ yeast extract, 5 g L^−1^ glycerol, 2.31 g L^−1^ KH_2_PO_4_, 12.54 g L^−1^ K_2_HPO_4_) grown at 22 °C and 200 rpm for 24 h. Expression was induced by addition of 1 mM IPTG once an OD_600_>0.6 was reached. Cells were harvest by centrifugation (3488 g, 20 min, 4 °C), cell pellets were washed once with 50 mM Tris⋅SO4 buffer, pH 7, centrifuged again and stored at −20 °C until further use.

Purification of HheG, HheG2 and HheG3 was carried out by affinity chromatography on an Äkta pure FPLC (GE Healthcare Life Sciences, Freiburg, Germany) as described previously.[^22^, ^24^] Briefly, cells were disrupted by suspension in binding buffer (50 mM Tris⋅SO_4_, 300 mM Na_2_SO_4_, 25 mM imidazole, pH 7.9) containing 1 mg mL^−1^ lysozyme and 100 μM phenylmethylsulfonyl fluoride and sonication (5 min of 10 s pulses at 65 % amplitude and 20 s min, on ice). The resulting crude lysate was cleared by centrifugation (16600 g, 30 min, 4 °C) and filtration (0.45 μm cellulose acetate membrane filter) and injected into an Äkta pure FPLC system equipped with a 5 mL HisTrap FF column (GE Healthcare). The column was washed, and the target protein was eluted with a linear gradient from 25 to 500 mM imidazole through increasing addition of elution buffer (50 mM Tris⋅SO_4_, 300 mM Na_2_SO_4_, 400 mM imidazole, pH 7.9). Fractions containing pure target protein (as assessed by SDS PAGE) were combined and concentrated by centrifugation (Vivaspin, Sartorius, Göttingen, Germany, molecular weight cut‐off at 10 kDa). Afterwards, the protein was desalted into glycerol‐containing TE buffer (10 mM Tris⋅SO_4_, 4 mM EDTA, 10 % (v/v) glycerol, pH 7.9) using a PD‐10 desalting column (GE Healthcare).

**Dehalogenation reactions**: To examine the three G‐type HHDHs for dehalogenation activity, reactions were performed with selected chloroalcohols and monitored with a halide release assay.[^34^, ^35^] To this end, reactions were performed with 5 or 20 mM chloroalcohol and 25 or 100 μg mL^−1^ HHDH in 25 mM Tris ⋅ SO_4_ buffer at pH 7 and 30 °C in a total volume of 0.2–1.5 mL in glass vials (for exact conditions see Table S1 in the Supporting Information). As negative controls, reactions without enzyme were carried out in parallel. After 1, 2, 3 and 4 min 100 μL samples were taken and quenched in a 100 μL solution containing a 1 : 1 mixture of halide release assay solution I (0.25 M NH_4_Fe(SO_4_)_2_ in 9 M HNO_3_) and II (saturated Hg(SCN)_2_ solution in ethanol) in a 96‐well plate. The absorption at 460 nm was measured with a CLARIOstar 96‐well microplate reader (BMG Labtech, Ortenberg, Germany). Absorption differences were converted to reaction rates and specific activities through a standard curve obtained with chloride.

**Substrate screening for epoxide ring opening reactions**: Conversion of the cyclic epoxides **3**–**10** was first tested using azide as the nucleophile. The accepted epoxides **3**–**5**, including cyclohexene oxide (**1**) and (+)‐*trans*‐limonene oxide (*trans*‐**2**), were then tested with cyanate, cyanide, formate, nitrite and thiocyanate as nucleophiles. According to previous work on HheG,[^22^, ^24^] this pre‐screening was performed in small scale reactions in a total volume of 1.5 mL in 50 mM Tris⋅SO_4_ buffer, pH 7.0 (in case of cyanide pH 8.0), at room temperature with 40 mM nucleophile (azide, cyanide, cyanate, thiocyanate, formate or nitrite) using 200 μg or 500 μg of HheG, HheG2 or HheG3, respectively, and 20 mM of substrate **1**–**5** (from 1 M stock solutions in *i*PrOH). In case of reactions with azide, cyanate and nitrite as nucleophiles as well as reactions with cyclohexene oxide (**1**) and *trans*‐limonene oxide (*trans*‐**2**) with cyanide 200 μg enzyme was used, in case of all other reactions with cyanide, thiocyanate and formate, 500 μg enzyme was used. Respective negative control reactions without enzyme but only substrate with nucleophile were carried out in parallel. For GC analysis, samples of the reaction mixtures were quenched in an equal volume of methyl‐*tert*‐butylether. The samples were shaken vigorously and centrifuged for phase‐separation. The organic phase was subsequently dried over anhydrous MgSO_4_ and samples were injected into achiral GC for the quantification of product formation.

**Synthesis of β‐substituted alcohols**: Whole cell conversions were carried out using *E. coli* BL21 (DE3) gold cells that heterologously expressed HheG, HheG2 or HheG3. Cell suspensions were used that displayed an OD_600_ of 40 or 80 at room temperature and 900 rpm. In case of conversions of epoxides **3**–**5** with azide, reactions were carried out using *E. coli* BL21 (DE3) gold cells that expressed HheG in 15 mL 50 mM Tris/SO_4_ buffer, pH 7, 300 mM epoxide and 200 mM azide (both from stock solutions) for 42 h. In case of conversions of epoxides **1**, *trans*‐**2** and **3**–**5** with nucleophiles cyanide, cyanate, thiocyanate, formate and nitrite, reactions were carried out using *E. coli* BL21 (DE3) gold cells that expressed HheG2 (in case of epoxides *trans*‐**2** and **5** with cyanide) or HheG3 in 10 mL 50 mM Tris/SO_4_ buffer, pH 7, using 35–42 mM epoxide substrate and two equivalents of the respective nucleophile. After the reaction, the mixture was extracted three times using ethyl acetate. The combined organic layers were dried over MgSO_4_ and the solvent was evaporated *in vacuo*. The obtained crude products were analyzed via achiral GC to check for product formation and then purified using flash chromatography on silica gel (silica gel 60, particle size 0.040–0.063 mm, mesh 230–440 ASTM, Fluka). Technical‐grade solvents for chromatography were distilled before use. Thin layer chromatography (TLC) was performed using silica coated plates Polygram SIL G/UV254 (Macherey & Nagel). TLC plates were stained with KMnO_4_ (0.75 g KMnO_4_, 5 g K_2_CO_3_ and 0.75 mL 10 % NaOH in 100 mL water). Analytical standards of the diols (arising from hydrolysis of nitrite esters accessed by epoxide ring opening or by direct hydrolysis of the epoxides in water) were accessed by a similar procedure, using an HHDH and nitrite as the nucleophile. This primarily yielded the *cis*‐diols after workup and chromatography as described above. Analytical data of the obtained products are available from the Supporting Information.

## Conflict of interest

There are no conflicts to declare.

1

## Supporting information

As a service to our authors and readers, this journal provides supporting information supplied by the authors. Such materials are peer reviewed and may be re‐organized for online delivery, but are not copy‐edited or typeset. Technical support issues arising from supporting information (other than missing files) should be addressed to the authors.

Supporting Information

## Data Availability

All NMR and protein structural data pertaining to this publication are freely available from an externally hosted repository (https://doi.org/10.5281/zenodo.6785962). Further data are available from the authors upon reasonable request.

## References

[1] A. Schallmey , M. Schallmey , Appl. Microbiol. Biotechnol. 2016, 100, 7827–7839. 27502414 10.1007/s00253-016-7750-y PMC4989007

[2] D. B. Janssen , I. J. T. Dinkla , G. J. Poelarends , P. Terpstra , Environ. Microbiol. 2005, 7, 1868–1882. 16309386 10.1111/j.1462-2920.2005.00966.x

[3] G. Hasnaoui-Dijoux , M. M. Elenkov , J. H. Lutje Spelberg , B. Hauer , D. B. Janssen , ChemBioChem 2008, 9, 1048–1051. 18357593 10.1002/cbic.200700734

[4] R. J. Fox , S. C. Davis , E. C. Mundorff , L. M. Newman , V. Gavrilovic , S. K. Ma , L. M. Chung , C. Ching , S. Tam , S. Muley , J. Grate , J. Gruber , J. C. Whitman , R. A. Sheldon , G. W. Huisman , Nat. Biotechnol. 2007, 25, 338–344. 17322872 10.1038/nbt1286

[5] S. K. Ma , J. Gruber , C. Davis , L. Newman , D. Gray , A. Wang , J. Grate , G. W. Huisman , R. A. Sheldon , Green Chem. 2010, 12, 81–86.

[6] N.-W. Wan , Z.-Q. Liu , F. Xue , K. Huang , L.-J. Tang , Y.-G. Zheng , Appl. Microbiol. Biotechnol. 2015, 99, 4019–4029. 25805343 10.1007/s00253-015-6527-z

[7] P. Yao , L. Wang , J. Yuan , L. Cheng , R. Jia , M. Xie , J. Feng , M. Wang , Q. Wu , D. Zhu , ChemCatChem 2015, 7, 1438–1444.

[8] Y. Luo , Y. Chen , H. Ma , Z. Tian , Y. Zhang , J. Zhang , Sci. Rep. 2017, 7, 1–9.28127051

[9] A. Sharma , J. Agarwal , R. K. Peddinti , Org. Biomol. Chem. 2017, 15, 1913–1920. 28169381 10.1039/c6ob02479c

[10] Q. Xu , K.-S. Huang , Y.-F. Wang , H.-H. Wang , B.-D. Cui , W.-Y. Han , Y.-Z. Chen , N.-W. Wan , ACS Catal. 2022, 12, 6285–6293.

[11] I. Dokli , N. Milčić , P. Marin , M. S. Miklenić , M. Sudar , L. Tang , Z. F. Blažević , M. M. Elenkov , Catal. Commun. 2021, 152, 106285.

[12] Z. Findrik Blažević , N. Milčić , M. Sudar , M. Majerić Elenkov , Adv. Synth. Catal. 2021, 363, 388–410.

[13] N. Wan , J. Tian , X. Zhou , H. Wang , B. Cui , W. Han , Y. Chen , Adv. Synth. Catal. 2019, 361, 4651–4655.

[14] M. Majerić Elenkov , L. Tang , A. Meetsma , B. Hauer , D. B. Janssen , Org. Lett. 2008, 10, 2417–2420. 18491860 10.1021/ol800698t

[15] M. Majerić Elenkov , W. H. Hoeffken , L. Tang , B. Hauer , D. B. Janssen , Adv. Synth. Catal. 2007, 349, 2279–2285.

[16] J. H. Lutje Spelberg , J. E. T. Van Hylckama Vlieg , T. Bosma , R. M. Kellogg , D. B. Janssen , Tetrahedron: Asymmetry 1999, 10, 2863–2870.

[17] M. Majerić Elenkov , I. Primožič , T. Hrenar , A. Smolko , I. Dokli , B. Salopek-Sondi , L. Tang , Org. Biomol. Chem. 2012, 10, 5063–5072. 22622806 10.1039/c2ob25470k

[18] F.-R. Zhang , N.-W. Wan , J.-M. Ma , B.-D. Cui , W.-Y. Han , Y.-Z. Chen , ACS Catal. 2021, 11, 9066–9072.

[19] M. An , W. Liu , X. Zhou , R. Ma , H. Wang , B. Cui , W. Han , N. Wan , Y. Chen , RSC Adv. 2019, 9, 16418–16422. 35516406 10.1039/c9ra03774h PMC9064361

[20] M. M. Elenkov , B. Hauer , D. B. Janssen , Adv. Synth. Catal. 2006, 348, 579–585.

[21] E. Calderini , J. Wessel , P. Süss , P. Schrepfer , R. Wardenga , A. Schallmey , ChemCatChem 2019, 11, 1–9.

[22] J. Koopmeiners , C. Diederich , J. Solarczek , H. Voß , J. Mayer , W. Blankenfeldt , A. Schallmey , ACS Catal. 2017, 7, 6877–6886.

[23] J. Koopmeiners , B. Halmschlag , M. Schallmey , A. Schallmey , Appl. Microbiol. Biotechnol. 2016, 100, 7517–7527. 27052376 10.1007/s00253-016-7493-9

[24] J. Solarczek , T. Klünemann , F. Brandt , P. Schrepfer , M. Wolter , C. R. Jacob , W. Blankenfeldt , A. Schallmey , Sci. Rep. 2019, 9, 1–10. 30911023 10.1038/s41598-019-41498-2 PMC6434027

[25] M. Staar , S. Henke , W. Blankenfeldt , A. Schallmey , ChemCatChem 2022, 14, e202200145.

[26] J. E. T. van Hylckama Vlieg , L. Tang , J. H. Lutje Spelberg , T. Smilda , G. J. Poelarends , T. Bosma , A. E. J. van Merode , M. W. Fraaije , D. B. Janssen , J. Bacteriol. 2001, 183, 5058–5066. 11489858 10.1128/JB.183.17.5058-5066.2001 PMC95381

[27] K. L. Kavanagh , H. Jörnvall , B. Persson , U. Oppermann , Cell. Mol. Life Sci. 2008, 65, 3895–3906. 19011750 10.1007/s00018-008-8588-y PMC2792337

[28] M. Schallmey , J. Koopmeiners , E. Wells , R. Wardenga , A. Schallmey , Appl. Environ. Microbiol. 2014, 80, 7303–7315. 25239895 10.1128/AEM.01985-14 PMC4249193

[29] J. Jumper , R. Evans , A. Pritzel , T. Green , M. Figurnov , O. Ronneberger , K. Tunyasuvunakool , R. Bates , A. Žídek , A. Potapenko , A. Bridgland , C. Meyer , S. A. A. Kohl , A. J. Ballard , A. Cowie , B. Romera-Paredes , S. Nikolov , R. Jain , J. Adler , T. Back , S. Petersen , D. Reiman , E. Clancy , M. Zielinski , M. Steinegger , M. Pacholska , T. Berghammer , S. Bodenstein , D. Silver , O. Vinyals , A. W. Senior , K. Kavukcuoglu , P. Kohli , D. Hassabis , Nature 2021, 596, 583–589. 34265844 10.1038/s41586-021-03819-2 PMC8371605

[30] M. Mirdita , K. Schütze , Y. Moriwaki , L. Heo , S. Ovchinnikov , M. Steinegger , Nat. Methods 2022, 19, 679–682. 35637307 10.1038/s41592-022-01488-1 PMC9184281

[31] H. Arabnejad , M. Dal Lago , P. A. Jekel , R. J. Floor , A.-M. W. H. Thunnissen , A. C. Terwisscha van Scheltinga , H. J. Wijma , D. B. Janssen , Protein Eng. Des. Sel. 2017, 30, 175–189. 10.1093/protein/gzw068 27999093

[32] H. Wang , N. Wan , R. Miao , C. He , Y. Chen , Z. Liu , Y. Zheng , Angew. Chem. 2022, 202205790, DOI: 10.1002/ange.202205790. 35856897

[33] S. L. Lovelock , R. Crawshaw , S. Basler , C. Levy , D. Baker , D. Hilvert , A. P. Green , Nature 2022, 606, 49–58. 35650353 10.1038/s41586-022-04456-z

[34] M. Schallmey , P. A. Jekel , L. Tang , M. Majerić Elenkov , H. W. Höffken , B. Hauer , D. B. Janssen , Enzyme Microb. Technol. 2015, 70, 50–57. 25659632 10.1016/j.enzmictec.2014.12.009

[35] J. G. Bergmann , J. Sanik , Anal. Chem. 1957, 29, 241–243.

