# Supplementary material for: G‐type Halohydrin Dehalogenases Catalyze Ring Opening Reactions of Cyclic Epoxides with Diverse Anionic Nucleophiles
Source: Chemistry. 2022 Nov 14;28(72):e202202343. doi: 10.1002/chem.202202343 (PMC10099379; doi:10.1002/chem.202202343)
Supplement: Supplementary file 1 — Supporting Information [file CHEM-28-0-s001.pdf]

Chemistry–A European Journal

Supporting Information

G-type Halohydrin Dehalogenases Catalyze Ring Opening Reactions of Cyclic Epoxides with Diverse Anionic Nucleophiles

Jennifer Solarczek, Felix Kaspar, Pia Bauer, and Anett Schallmey*

Table of Contents

Author contributions S2

Accessibility statement S2

General remarks S2

Database mining S3

Enzymes S3

Experimental details, supplementary items, and additional discussion S4

- Structure prediction *via* AlphaFold2 S4
- Cloning S6
- Protein expression and purification S6
- Dehalogenation reactions and halide release assay S7
- Unfolding thermodynamics S8
- Substrate screening S11
- Synthesis of β-substituted alcohols by HHDH-mediated epoxide opening S11

NMR data S20

Achiral and chiral GC chromatograms S20

Supplementary references S35

Author contributions (with definitions as recommended by Brand *et al.*[1])

Conceptualization, A.S.; Data curation, J.S., and F.K.; Formal analysis, J.S., and F.K.; Funding acquisition, A.S.; Investigation, J.S., F.K. and P.B.; Methodology, J.S., and F.K.; Project administration, A.S.; Resources, A.S.; Software, - ; Supervision, A.S.; Validation, - ; Visualization, F.K.; Writing—original draft, F.K.; Writing—review & editing, all authors.

Accessibility statement

The data presented in this manuscript are depicted using scientific color maps. Since ca. 4% of the human population are color vision-deficient, we make a conscious effort to avoid unscientific uses of color, such as color ambiguities and other biases that could lead to misrepresentation, limited accessibility, or loss of information upon reduction of the color space.[2] Thus, (with one exception) all data in this manuscript were color-coded in the scientific color map *plasma*.

General remarks

All chemicals used in this study were of analytical grade or higher and purchased from Sigma Aldrich (Steinheim, Germany), Fisher Scientific (Schwerte, Germany), Carl Roth (Karlsruhe, Germany), TCI Deutschland (Eschborn, Germany) or VWR (Darmstadt, Germany) and used without prior purification. Deionized water was used for the preparation of all enzymatic reactions as well as purification and storage buffers. Enzymatic reactions were generally prepared from stock solutions of substrate(s), salt(s), buffer(s) and enzyme(s) and started *via* the addition of enzyme or key reagent as indicated for the respective experiment. Protein concentrations were determined with a NP80 nanophotometer (Implen, Munich, Germany) and melting points of proteins were determined with a QuantStudio1 Real-Time-PCR System (Thermo Fisher Scientific, Waltham, USA). Extinction coefficients of proteins were estimated with Protparam.[3] NMR spectra were recorded on a Bruker AV300, Bruker AVIII400, Bruker AVIIIHD500 (with CryoProbe) and Bruker AVANCE III 600 instrument using the solvent peaks as internal reference. The following abbreviations were used for ^{1}H and ^{13}C NMR chemical shifts: s = singlet, d = doublet, t = triplet, q = quartet, m = multiplet and combinations thereof. GC-MS was performed using an Agilent 6890 gas chromatograph equipped with a 30 m analytical column (Phenomenex ZB1-MS, 30m x 0.25 mm ID, t_f=0.25 µm). A split injection port at 270°C was used for sample introduction and the split ratio was set to 10:1. The used temperature program was 50°C (3min)-10°C/min-310°C (3min). The helium carrier gas was set to 1.0 ml/minute flow rate (constant flow mode). The transfer line was kept at 270°C. A JMS-T100GC (GCAccuTOF, JEOL, Japan) time of flight mass spectrometer in electron ionization (EI) mode at 70eV and JEOL MassCenter™ workstation software was used. The source and transfer line temperature were set at 200°C and 270°C respectively. The detector voltage was set at 2000V. The acquisition range was from m/z 41 to 600 with spectrum recoding interval of 0.4 s. The system was tuned with PFK to achieve a resolution of 5,000 (FWHM) at m/z 292.9824. The conversion determination as well as the separation of enantiomers and determination of enantiomeric excess of the reactions were carried out using a GC2010 plus gas chromatograph equipped with an FID detector. For the determination of the conversion and detection of product formation, the achiral column Optima 5 MS with a length of 30 m, an inner diameter of 0.25 mm, a film thickness of 25 µm and a flow of 1.24 mL min 1 was employed. For the separation of enantiomers, the chiral column HYDRODEX γ-DIMOM with a length of 25 m, an inner diameter of 0.25 mm, a film thickness of 0.25 µm and a flow of 1.2 mL min^{-1} was used. The temperature programs for achiral analysis were: method A) 110 °C, 7.5 min; 50 °C min^{-1}, 295 °C, 2 min, method B) 100 °C, 3 min; 50 °C min^{-1}, 200 °C; 20 °C min^{-1}; 300 °C, 2.5 min, method C) 50 °C, 3 min; 20 °C min^{-1}, 340 °C, 2.5 min and method D) 80 °C, 1 min; 10 °C min^{-1}, 160 °C; 20 °C min^{-1}, 300 °C. Temperature programs for chiral analysis were: method A) 40 °C; 10 °C min^{-1}; 90 °C, 20 min; 5 °C min^{-1}; 220 °C and method B) 100 °C, 40 min; 10 °C min^{-1}; 200 °C, 2 min. Data handling and routine calculations were carried out in Excel or LibreOffice, NMR analysis in MestreNova, protein structure prediction *via* the ColabFold implementation of AlphaFold2 (see below),[4] and analysis, fitting and data plotting in OriginLab (2020b or 2021 version).

Database mining

A PHI-BLAST[5] search in the nr database of GenBank (release 230) was performed using the protein sequence of HheG as query in combination with our previously defined HHDH-specific sequence motifs 1 (T-X_4-F/Y-X-G) and 2 (S-X_{12}-Y-X_3-R).[6] A multiple sequence alignment of the resulting homologous sequences was generated with the MAFFT webserver to infer the probable phylogenetic relationship based on average linkage (UPGMA).[7] This identified a hypothetical protein from *Actinobacteria bacterium* (GenBank accession number: RLE25108) as a new member of G-type HHDHs and was therefore named HheG3. The gene of HheG3 was ordered as synthetic gene from GeneArt (Thermo Fisher Scientific, Regensburg, Germany) with codon optimization for *Escherichia coli*.

Enzymes

HheG (*Ilumatobacter coccineus* YM16-304; Uniprot A0A6C7EF96; Accession number BAN3849, ε_{280} = 15470 M^{-1} cm^{-1})
MGSSHHHHHHSSGLVPRGSHMSNAENRPVALITMATGYVGPALARTMADRGFDLVLHGTAGDGTMVGVEESFD
SQIADLAKRGADVLTISDVDLTTRTGNQSMIERVLERFGRLDSACLVTGLIVTGKFLDMTDDQWAKVKATNLDMVF
HGLQAVLPPMVAAGAGQCVVFTSATGGRPDPMVSIYGGTRAGANGIVRAVGLEHARHGVQVNAIGTNYMDFPG
FLKASRADGDPERRAMIEAQVPLRRLGTMDELSSVTAGLLDGSNRFQTGQFFDFSGGWGA

HheG2 (*Ilumatobacter nonamiensis*; Accession number WP_040495182.1, ε_{280} = 15470 M^{-1} cm^{-1})
MGSSHHHHHHSSGLVPRGSHMPSNERPVALITMATGYVGPALARTLAGRGYDLVLQGAAGDDSMVGVERSFET
QVPVLEALGAAVETVTDVDLRTAEGNRAVVQAALDRFGRLDSACFVTGVIIVGKFLDMSSEHWDQIKRNNLDMVF
HALQATLPPMVDAGAGQVVVFTSATGGRPDPMTSIYGGTRAGANGIVRAVGLEHARDGVQVNAVGTNFMDFPG
FIKASGADDPERRKRIESQTPMRRLGTMDELANVTAVLLDGTNRFQTGQFFDFSGGWGA

HheG3 (*Actinobacteria bacterium*; Accession number RLE25108, ε_{280} = 16960 M^{-1} cm^{-1})
MGSSHHHHHHSSGLVPRGSHMSETRPTALVTMATGYLGPALALNLASKGFDLVLQGMADSNNMVGVEVPFSEL
VPELESTGVAVHTVTDVDLSTAEGNQSLVAQAIDRFGRLDSACYVTGMIVVGKFLDMTTKQWETVKRMNLDMVF
HGLQAALPPMVEAGAGQIVVFTSATGARPEPMVSIYGGTRAGANGLIRAVGLEHAADGIQVNAVGTNYMDFPGF
MKASGADSSPERRAAIEAQVPMKKLGTMEELAAFTSVLLDGSSRFQTGQFFSFSGGWST

Experimental details, supplementary items, and additional discussion

Structure prediction *via* AlphaFold2

To compare the likely tertiary structures of HheG, HheG2 and HheG3, structure predictions with AlphaFold2 were performed, as an experimental crystal structure is currently only available for HheG and three of its mutants. Since all three HHDHs in this study display relatively high sequence identity (≥70%) and their protein sequences generally align well (Figure S1A), we anticipated a similar overall protein structure. To examine this hypothesis, we obtained structural predictions for the three proteins *via* the AlphaFold2 (AF2) implementation ColabFold.[4] Therefore, the entire protein sequences (PDB ID 5o30 for HheG, Accession number WP_040495182.1 for HheG2 and Accession number RLE25108 for HheG3) were used as query sequences, although the C-terminus only adopts a defined structure about 10 amino acids into the protein. The default settings of ColabFold (version 1.2.0, msa_mode MMseqs2, model_type auto, 3 recycles, prediction of the monomeric structure) were used to obtain the five best ranking models by pLDDT. These models compare well to the crystal structure of HheG, except for selected loop regions. In particular, the loop region from Gly38 to Ser51 (following the numbering of 5o30) was predicted with very low confidence and significant flexibility. The reason for this heterogeneity is the fact that this loop region is truly unique to the G-type family. AF2's sequence search identified only very few similar sequences for this loop region and essentially none that cover it start-to-end (Figure S1B). In contrast, the backbone of all three enzymes is highly similar to related short-chain dehydrogenases/reductases and could be predicted with high confidence in the conserved fold. Based on these findings, we were confident that the three enzymes possess a very similar tertiary structure and essentially identical active site structures. All prediction results are available from the externally hosted supplementary information.[8]

A

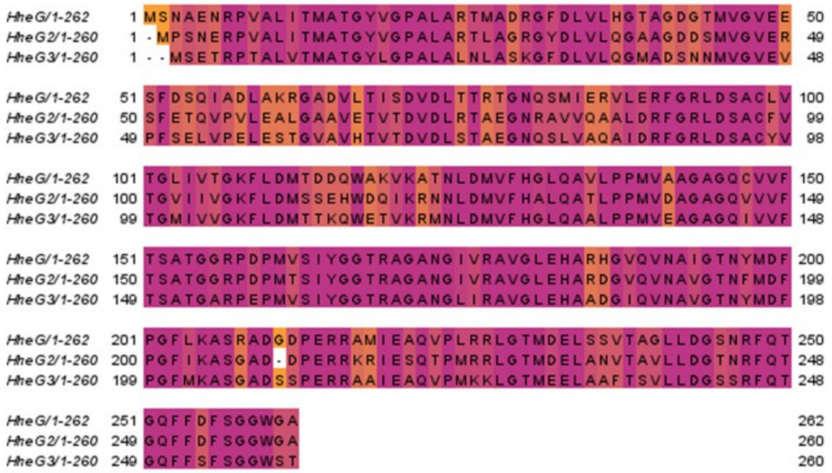

B

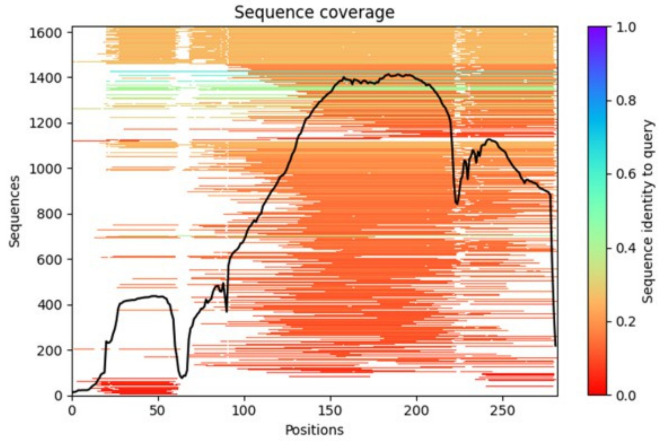

Figure S1. A Multiple sequence alignment of HheG, HheG2 and HheG3 (the gradient from yellow to purple indicates low to high conservation) and **B** sequence coverage of HheG in AF2's database search. **A** was obtained from alignment of the three sequences *via* ClustralO and **B** is an output file of ColabFold.

Cloning

Expression plasmids for HheG and HheG2 were available from previous studies.[9,10]

The synthetic gene of HheG3 was excised from the pMA-RQ-RLE25108 vector with the endonucleases HindIII-HF and NdeI, followed by ligation with T4 DNA ligase (all DNA-modifying enzymes were purchased from New England BioLabs, Frankfurt, Germany) into the linearized expression vector pET28a(+). After transformation of *E. coli* XL-1 blue cells with the ligation construct, recombinant plasmid DNA was isolated *via* the E.Z.N.A. Plasmid Mini Kit I (Omega Bio-Tek Inc, Norcross, USA).

Protein expression and purification

After transformation of *E. coli* BL21 (DE3) gold cells with the respective plasmid, heterologous enzyme production of halohydrin dehalogenase HheG, HheG2 as well as HheG3 was carried out as reported previously.[9,10] Briefly, a preculture of the respective expression strain was grown in LB medium (10 g L^{-1} tryptone, 5 g L^{-1} yeast extract, 10 g L^{-1} NaCl) overnight, which was used inoculate an expression culture in TB medium (12 g L^{-1} tryptone, 24 g L^{-1} yeast extract, 5 g L^{-1} glycerol, 2.31 g L^{-1} KH_2PO_4, 12.54 g L^{-1} K_2HPO_4) grown at 22 °C and 200 rpm for 24 h. Expression was induced by addition of 1 mM IPTG once an OD_{600} > 0.6 was reached. Cells were harvest by centrifugation (3488 g, 20 min, 4 °C), cell pellets were washed once with 50 mM Tris·SO4 buffer, pH 7, centrifuged again and stored at −20°C until further use.

Purification of HheG, HheG2 and HheG3 was carried out by affinity chromatography on an Äkta pure FPLC (GE Healthcare Life Sciences, Freiburg, Germany) as described previously.[9,10] Briefly, cells were disrupted by suspension in binding buffer (50 mM Tris·SO_4, 300 mM Na_2SO_4, 25 mM imidazole, pH 7.9) containing 1 mg mL^{-1} lysozyme and 100 µM phenylmethylsulfonyl fluoride and sonication (5 min of 10 s pulses at 65% amplitude and 20 s min, on ice). The resulting crude lysate was cleared by centrifugation (16600 g, 30 min, 4 °C) and filtration (0.45 µm cellulose acetate membrane filter) and injected into an Äkta pure FPLC system equipped with a 5 mL HisTrap FF column (GE Healthcare). The column was washed, and the target protein was eluted with a linear gradient from 25 to 500 mM imidazole through increasing addition of elution buffer (50 mM Tris·SO_4, 300 mM Na_2SO_4, 400 mM imidazole, pH 7.9). Fractions containing pure target protein (as assessed by SDS PAGE) were combined and concentrated by centrifugation (Vivaspin, Sartorius, Göttingen, Germany, molecular weight cut-off at 10 kDa). Afterwards, the protein was desalted into glycerol-containing TE buffer (10 mM Tris·SO_4, 4 mM EDTA, 10% (v/v) glycerol, pH 7.9) using a PD-10 desalting column (GE Healthcare). Protein concentrations were determined with a NP80 nanophotometer (Implen, Munich, Germany) using the extinction coefficients listed on page S3. Typical stock concentrations ranged from 7−17 g L^{-1} (calculated *via* the A_{280}, considering the molecular weight of the respective protein). This procedure typically yielded around 80−150 mg L^{-1} pure protein for HheG, around 120 mg L^{-1} for HheG2 and around 50 mg L^{-1} for HheG3.

Dehalogenation reactions and halide release assay

To examine the three G-type HHDHs for dehalogenation activity, reactions were performed with selected chloroalcohols and monitored with a halide release assay.[11,12] To this end, reactions were performed with 5 or 20 mM chloroalcohol (**S1**−**S4**) and 25 or 100 µg mL^{-1} HHDH in 25 mM Tris·SO_4 buffer at pH 7 and 30 °C in a total volume of 0.2−1.5 mL in glass vials (for exact conditions see Table S1 below). As negative controls, reactions without enzyme were carried out in parallel. After 1, 2, 3 and 4 min 100 µL samples were taken and quenched in a 100 µL solution containing a 1:1 mixture of halide release assay solution I (0.25 M $NH_4Fe(SO_4)_2$ in 9 M HNO_3) and II (saturated $Hg(SCN)_2$ solution in ethanol) in a 96-well plate. The absorption at 460 nm was measured with a CLARIOstar 96-well microplate reader (BMG Labtech, Ortenberg, Germany). Absorption differences were converted to reaction rates and specific activities through a standard curve obtained with chloride.

Table S1. Reaction conditions for HHDH-catalyzed dehalogenation of chloroalcohols

	HheG		HheG2	HheG3
Substrate	**S1**, **S2**, **S3**	**S4**	**S1**−**S4**	**S1**−**S4**
Substrate (mM)	5	5	5	20
Reaction volume (mL)	0.5	1.5	0.2	1.0
Enzyme (µg mL^{-1})	25	100	25	100

Substrates

S1
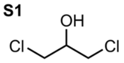

S2
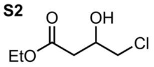

S3
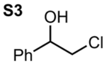

S4
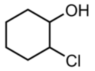

Unfolding thermodynamics

To investigate the stability of the HHDHs as well as their cosolvent resistance, differential scanning fluorimetry experiments were performed. To this end, HHDHs (0.2 g L^{-1}) were incubated with SYPRO orange (5x) in TE buffer buffer (10 mM Tris·SO_4, 4 mM EDTA, pH 7.9) containing 3% (v/v) glycerol and 0−4% (v/v) *i*PrOH in a total volume of 25 µL in a sealed well of a PCR plate, in analogy to our previous reports.[10,13] These solutions were incubated at 10 °C for 3 min and subsequently heated in steps of 0.5 °C per 15 s to a final temperature of 90 °C in a QuantStudio1 Real-Time-PCR System (Thermo Fisher Scientific, Waltham, USA) system. At each temperature interval, fluorescence was measured (λ_{ex} = 470 nm, λ_{em} = 570 nm). The melting point T_m was then determined as the inflection point of the fluorescence intensity over the temperature (equal to the extreme of the first derivative of the fluorescence). This fluorescence data yielded the unfolding thermodynamics *via* equations reported by Konkolewicz and Page and colleagues.[14] First, the raw fluorescence data was used to obtain the fraction of folded protein P_f as a function of the incubation temperature, while assuming a simplified two-state model of protein (un-)folding and a symmetrical melting curve.

$$P_f = \frac{F - F_{min}}{F_{max} - F_{min}} \tag{S1}$$

where

$$F_{max} = F_{Tm} - F_{min} + F_{Tm} \tag{S2}$$

which can be simplified to

$$P_f = \frac{F - F_{min}}{2F_{Tm} - 2F_{min}} \tag{S3}$$

where P_f is the fraction of folded protein, F is the observed fluorescence as a function of the incubation temperature, F_{min} is the minimum of the observed fluorescence (generally 5−10 K below the melting point T_m), F_{max} is the maximum of the fluorescence, which is assumed to follow in symmetry to the minimum by approximation via F_{Tm}, the fluorescence at the melting point. All of these variables are dimensionless or are given in arbitrary (or relative) fluorescence units. From the results of these equations, the fraction of unfolded protein can be obtained through the corresponding mass balance from which the equilibrium constant of unfolding follows as given by the law of mass action.

$$P_u = 1 - P_f \tag{S4}$$

$$K_u = \frac{P_u}{P_f} = \frac{1 - P_f}{P_f} \tag{S5}$$

where P_u is fraction of unfolded protein and K_u is the equilibrium constant of unfolding (both also dimensionless). This provides access to the thermodynamic properties of unfolding in an Arrhenius fashion by fitting with the linear equation (S6) and application of (S7).

$$lnK_u = \frac{-\Delta H_u^0 + T\,\Delta S_u^0}{RT} = -\frac{\Delta H_u^0}{R}\frac{1}{T} + \frac{\Delta S_u^0}{R} \tag{S6}$$

$$\Delta G_u^0 = \Delta H_u^0 - T\,\Delta S_u^0 \tag{S7}$$

where ΔG_u^0 is the apparent standard Gibbs fee energy of unfolding (kJ mol^{-1}), ΔH_u^0 the apparent standard enthalpy of unfolding (kJ mol^{-1}), ΔS_u^0 the apparent standard entropy of unfolding (kJ mol^{-1} K^{-1}), R is the universal gas constant (8.314 J mol^{-1} K^{-1}) and T is the temperature (K). For this calculation, we generally only considered data points corresponding to unfolding percentages of P_u = 0.1−0.5, which corresponds to the initial slope of the melting curve toward T_m. Thus, regions of the data space where either very little unfolded protein is present (P_u << 0.1) or where unspecific effects such as aggregation and higher order (un-)folding phenomena deviating from the assumed two-state model dominate (P_u > 0.5) are excluded.

Representative melting curves are shown below in Figure S2. Generally, the assumptions of simple two-state unfolding (*via* data treatment according to equations (S1)−(S7)) holds up remarkably well for the studied HHDHs. While HheG and HheG gave essentially linear Arrhenius plots for the considered data space (agreeing with the underlying mathematical assumptions), HheG showed noticeable curvature arising from the presence of a small unfolding peak prior to the T_m. Although the presence of a small quantity of contaminating (copurified) protein would be a likely explanation for this behavior, we attribute this curve shape to partial unfolding of the protein tetramer (G-type HHDHs are dimers of dimers) and binding of the fluorescent dye to the dimer interface.

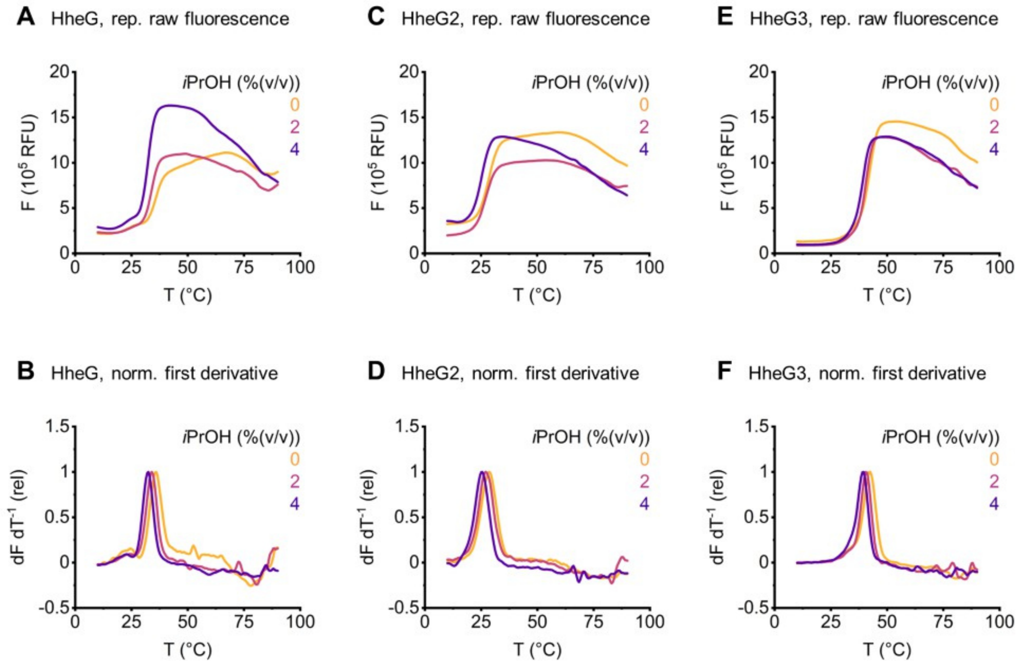

Figure S2. Representative melting curves of the three HHDHs.

To estimate the half-lives of the studied HHDHs, the decrease of their activity was assessed as a function of incubation time in reaction buffer. To this end, the HHDHs (13.33 µg mL^{-1}) were incubated in 50 mM Tris buffer pH 7 containing 40 mM azide in a total volume of 1.470 mL. After 0 and 6 h (0 and 2 h for HheG2), the activity of the enzymes was assessed by starting azidolysis reactions with cyclohexene oxide (1). Therefore, 30 µL cyclohexene oxide from a 1 M stock solution in iPrOH was added.

After 2, 4.5, 8 and 15 min, samples of 400 µL were taken from the reaction mixture and quenched in an equal volume of methyl-*tert*-butylether (MTBE). The samples were shaken vigorously and centrifuged for phase-separation. The organic phase was subsequently dried over anhydrous $MgSO_4$ and samples were injected into achiral GC for the quantification of product formation. The reaction rate was approximated as linear (zero order assumptions) and the decrease of the resulting enzymatic activities was fitted as a first-order exponential decay according to equation (S8), yielding estimated half-lives *via* equation (S9).

$$a(t) = a_0 \exp\left(-\frac{t}{\tau}\right) \quad \text{(S8)}$$

$$t_{1/2} = \tau\, ln(2) \quad \text{(S9)}$$

where a is the activity of the HHDH (U mg^{-1}) with one unit (U) of activity corresponding to the formation of one µmol of product (azidoalcohol **1a** in this case) per minute under the described reaction conditions, a_0 is the initial activity (before incubation; also U mg^{-1}), t is the incubation time (h), τ is the mean lifetime of the enzyme (h) and $t_{1/2}$ is the half-life (h).

Substrate screening

Conversion of the cyclic epoxides **3**–**10** was first tested using azide as the nucleophile. The accepted epoxides **3**–**5**, including cyclohexene oxide (**1**) and (+)-*trans*-limonene oxide (*trans*-**2**), were then tested with cyanate, cyanide, formate, nitrite and thiocyanate as nucleophiles.

According to previous work on HheG,[9,10] this pre-screening was performed in small scale reactions in a total volume of 1.5 mL in 50 mM Tris·SO_4 buffer, pH 7.0 (in case of cyanide pH 8.0), at room temperature with 40 mM nucleophile (azide, cyanide, cyanate, thiocyanate, formate or nitrite) using 200 µg or 500 µg of HheG, HheG2 or HheG3, respectively, and 20 mM of substrate **1**–**5** (from 1 M stock solutions in *i*PrOH). In case of reactions with azide, cyanate and nitrite as nucleophiles as well as reactions with cyclohexene oxide (**1**) and *trans*-limonene oxide (*trans*-**2**) with cyanide 200 µg enzyme was used, in case of all other reactions with cyanide, thiocyanate and formate, 500 µg enzyme was used. Respective negative control reactions without enzyme but only substrate with nucleophile were carried out in parallel. Sample preparation for GC analysis was carried out as described above.

Synthesis of β-substituted alcohols by HHDH-mediated epoxide opening

For the synthesis of the β-substituted alcohols on preparative-scale for structure validation, whole cell conversions were carried out using *E. coli* BL21 (DE3) gold cells that heterologously expressed HheG, HheG2 or HheG3. Cell suspensions were used that displayed an OD_{600} of 40 or 80 in the respective volumes stated below at room temperature and 900 rpm for the times indicated. In case of conversions of epoxides **3**–**5** with azide, reactions were carried out using *E. coli* BL21 (DE3) gold cells that expressed HheG in 15 mL 50 mM Tris/SO_4 buffer, pH 7, 300 mM epoxide and 200 mM azide (both from stock solutions) for 42 h. In case of conversions of epoxides **1,** *trans*-**2** and **3**–**5** with nucleophiles cyanide, cyanate, thiocyanate, formate and nitrite, reactions were carried out using *E. coli* BL21 (DE3) gold cells that expressed HheG2 (in case of epoxides *trans*-**2** and **5** with cyanide) or HheG3 in 10 mL 50 mM Tris/SO_4 buffer, pH 7, using 35–42 mM epoxide substrate and two equivalents of the respective nucleophile. After the reaction, the mixture was extracted three times using ethyl acetate. The combined organic layers were dried over $MgSO_4$ and the solvent was evaporated *in vacuo*. The obtained crude products were analyzed *via* achiral GC to check for product formation and then purified using flash chromatography on silica gel (silica gel 60, particle size 0.040-0.063 mm, mesh 230–440 ASTM, Fluka). Technical-grade solvents for chromatography were distilled before use. Thin layer chromatography (TLC) was performed using silica coated plates Polygram SIL G/UV254 (Macherey & Nagel). TLC plates were stained with $KMnO_4$ (0.75 g $KMnO_4$, 5 g K_2CO_3 and 0.75 mL 10% NaOH in 100 mL water).

Analytical standards of the diols (arising from hydrolysis of nitrite esters accessed by epoxide ring opening or by direct hydrolysis of the epoxides in water) were accessed by a similar procedure, using an HHDH and nitrite as the nucleophile. This primarily yielded the *cis*-diols after workup and chromatography as described above.

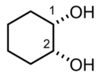

1f*

cis-Cyclohexane-1,2-diol (**S5**/**1f***)

Following the general procedure with **1**, nitrite and HheG (OD_{600}=40, 40 h reaction time), diol **S5** was obtained as a yellowish solid (10.7 mg, 13%, R_f = 0.30 in EtOAc). **^{1}H NMR** (400 MHz, CD_3OD): δ (ppm) 3.27 (2H, m, H-1 and H-2), 1.91 (2H, m, H-3 and H-6), 1.73–1.63 (2H, m, H-3 and H-6), 1.27 (4H, m, H-4 and H-5). **^{13}C NMR** (101 MHz, CD_3OD): δ (ppm) 76.17 (CH, C-1 and C-2), 34.11 (CH_2, C-3 and C-6), 25.33 (CH_2, C-4 and C-5). **EI-HRMS** m/z $[M]^+$ calc. for $C_6H_{12}O_2$ 116.08373, found 116.08318. **Achiral GC** 3.4 min (method A), 4.5 min (method B).

(1*S*,2*S*,4*R*)-1-methyl-4-(prop-1-en-2-yl)cyclohexane-1,2-diol (**S6/2f***)

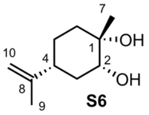

S6

Following the general procedure with *trans*-**2**, nitrite and HheG3 (OD_{600}=40, 19 h reaction time), diol **S6** was obtained as a colorless oil (23.2 mg, 55%, R_f = 0.04 in 10:1 pentane:EtOAc).
^{1}H NMR (400 MHz, $CDCl_3$) δ (ppm) 4.73 (2H, m, H-10), 3.67–3.60 (1H, m, H-2), 2.27 (1H, dddd, *J* = 7.7, 7.2, 2.9 Hz, H-4), 1.93 (1H, ddd, *J* = 13.9, 11.8, 2.9 Hz, H-3), 1.81–1.71 (4H, m, H-3 and H-9), 1.67 (1H, ddd, *J* = 13.8, 4.0, 1.1 Hz, H-5), 1.59–1.54 (3H, m, H-3 and H-5), 1.27 (3H, s, H-7). **^{13}C NMR** (101 MHz, $CDCl_3$) δ (ppm) 149.45 (Cq, C-8), 109.14 (CH_2, C-10), 74.10 (CH, C-2), 71.45 (CH, C-1), 37.62 (CH, C-4), 34.18 (CH_2 ,C-3), 33.84 (CH_2, C-6), 26.83 (CH_3, C-7), 26.35 (CH_2, C-5), 21.22 (CH_3, C-9). **EI-HRMS** m/z $[M]^+$ calc. for $C_{10}H_{18}O_2$ 170.13068, found 170.13197. **Achiral GC** 9.7 min (method D).

1-Methylcyclohexane-1,2-diol (**S7/4f***)

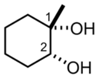

S7

Following the general procedure with *rac*-**4**, nitrite and HheG (OD_{600}=40, 40 h reaction time), diol **S7** was obtained as a colorless oil (13 mg, 17%, R_f = 0.29 in EtOAc). **^{1}H NMR** (400 MHz, $CDCl_3$) δ (ppm) 3.53–3.45 (1H, m, H-2), 1.92–1.78 (1H, m, H-6*), 1.78–1.66 (2H, m, H-3* and H-6*), 1.65–1.56 (1H, m, H-3*), 1.43–1.27 (4H, m, H-4 and H-5), 1.19 (3H, s, H-7). **^{13}C NMR** (101 MHz, $CDCl_3$): δ (ppm) 77.37 (CH, C-2), 74.10 (Cq, C-1), 38.77 (CH_2, C-6), 31.19 (CH_2, C-3), 24.18 (CH_2, C-4*), 23.41 (CH_2, C-5*), 19.77 (CH_3, C-7). *signals interchangeable. **EI-HRMS** m/z $[M]^+$ calc. for $C_7H_{14}O_2$ 130.0994, found 130.10237. **Achiral GC** 3.9 min (method A).

4-Vinylcyclohexane-1,2-diol (**S8/5f***)

S8

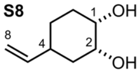

Following the general procedure with *rac*-**5**, nitrite and HheG (OD_{600}=40, 40 h reaction time), diol **S8** was obtained as a colorless oil (27 mg, 39%, R_f = 0.37 in EtOAc). **^{1}H NMR** (400 MHz, $CDCl_3$) δ (ppm) 5.84 (1H, ddd, *J* = 17.4, 10.6, 5.8 Hz, H-7), 5.08 (1H, ddd, *J* = 10.3, 1.6, 0.2 Hz, H-8), 5.04 (1H, ddd, *J* = 3.6, 1.6, 0.2 Hz, H-8), 3.61 (1H, ddd, *J* = 10.3, 8.0, 4.3 Hz, H-1), 3.42 (1H, ddd, *J* = 9.4, 7.9, 4.2 Hz, H-2), 2.58–2.50 (1H, m, H-4), 1.97 (1H, dddd, *J* = 13.1, 8.2, 4.2, 2.0 Hz, H-3), 1.86–1.76 (1H, m, H-6*), 1.76–1.66 (1H, m, H-3*), 1.62–1.45 (3H, m, H-5 and H-6*). **^{13}C NMR** (101 MHz, $CDCl_3$) δ (ppm) 141.42 (CH, C-7), 114.37 (CH_2, C-8), 75.22 (CH, C-1), 71.62 (CH, C-2), 36.28 (CH, C-4), 36.26 (CH_2, C-3), 28.37 (CH_2, C-6), 27.80 (CH_2, C-5). *signals interchangeable. **EI-HRMS** m/z $[M]^+$ calc. for $C_8H_{14}O_2$ 142.0994, found 142.10976. **Achiral GC** 7.4 min (method A) and 8.9 min (method C).

2-Azidocyclopentan-1-ol (**3a**)

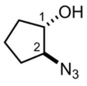

3a

Following the general procedure with **3**, azide and HheG (OD_{600}=40, 42 h reaction time), azido-alcohol **3a** was obtained as a colorless oil (146 mg, 52%, R_f = 0.37 in 5:1 pentane/EtOAc). **^{1}H NMR** (600 MHz, $CDCl_3$): δ (ppm) 4.06−4.02 (1H, m, H-1), 3.69−3.64 (1H, m, H-2), 2.08−2.02 (1H, m, H-3), 2.00−1.93 (1H, m, H-5), 1.82−1.52 (4H, m, H-3, H-5, and H-4). **^{13}C NMR** (151 MHz, $CDCl_3$): δ (ppm) 77.7 (CH, C-1), 68.8 (CH, C-2), 32.3 (CH_2, C-3), 28.7 (CH_2, C-4), 20.6 (CH_2, C-5). **EI-HRMS** m/z $[M+H]^+$ calc. for $C_5H_{10}N_3O$

128.0824, found 128.0829. **Achiral GC** 7.5 min (method C). **Chiral GC** 33.95 and 34.18 min (method A).

2-azido-1-methylcyclohexan-1-ol (**4a**) and 1-azido-1-methylcyclohexan-2-ol (**4a′**)

Following the general procedure with *rac*-**4**, azide and HheG (OD_{600}=40, 42 h reaction time), azido-alcohols **4a** and **4a′** were obtained as a mixture of regioisomers as a colorless oil (237 mg, 63%, R_f = 0.36 in 7:1 pentane/EtOAc).

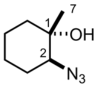

4a

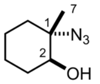

4a'

4a: **^{1}H NMR** (400 MHz, $CDCl_3$): δ 3.35 (1H, dd, *J* = 4.0, 10.5 Hz, H-2), 1.97−1.92 (1H, m, H-3), 1.86−1.29 (7H, m, H-3, H-4, H-5 and H-6), 1.18 (3H, s, H-7). **^{13}C NMR** (101 MHz, $CDCl_3$): δ 73.2 (Cq, C-1), 69.3 (CH, C-2), 38.4 (CH_2, C-3), 28.4 (CH_2, C-6), 23.9 (CH_2, C-4*), 22.9 (CH_2, C-5*), 21.9 (CH_3, C-7). *signals interchangeable. **EI-HRMS** m/z $[M+Na]^+$ calc. for $C_7H_{13}N_3O$ 178.0956, found 178.0952. **Achiral GC** 6.0 min (method A). **Chiral GC** 35.3 and 35.6 min (method A).

4a′: **^{1}H NMR** (400 MHz, $CDCl_3$): δ 3.48 (1H, dd, *J* = 4.0, 9.5 Hz, H-2), 1.86−1.29 (8H, m, H-3, H-4, H-5, and H-6), 1.28 (3H, s, H-7). **^{13}C NMR** (101 MHz, $CDCl_3$): δ 74.9 (Cq, C-1), 65.7 (CH, C-2), 35.0 (CH_2, C-3), 30.3 (CH_2, C-6), 23.2 (CH_2, C-4*), 22.2 (CH_2, C-5*), 16.9 (CH_3, C-7). *signals interchangeable. **EI-HRMS** m/z $[M+Na]^+$ calc. for $C_7H_{13}N_3O$ 178.0956, found 178.0952. **Achiral GC** 6.2 min (method A). **Chiral GC** 37.3 and 37.6 min (method A).

2-azido-4-vinylcyclohexan-1-ol (**5a**) and 1-azido-4-vinylcyclohexan-2-ol (**5a′**)

Following the general procedure with *rac*-**5**, azide and HheG (OD_{600}=40, 42 h reaction time), azido-alcohols **5a** and **5a′** were obtained as mixture of isomers as a colorless oil (243 mg, 70%, R_f = 0.38 in 8:1 pentane/EtOAc). Since **5a** and **5a′** proved inseparable by column chromatography and their mixture gave highly overlapping ^{1}H NMR signals, the NMR data listed here represent the sum of both compounds. **^{1}H NMR**[a] (400 MHz, $CDCl_3$): δ 5.83−5.72 (2H, m, H-7), 5.08−5.00 (4H, m, H-8), 3.71−3.65 (1H, ddd, *J* = 3.9, 7.5, 8.6 Hz, H-1*), 3.54−3.45 (2H, m, H-1* or H-2*), 3.36−3.31 (1H, m, H-2*), 2.54−2.43 (2H, m, H-4), 1.99−1.75 (4H, m, H-3 and H-6), 1.67−1.51 (8H, m, H-3, H-5, and H-6). **^{13}C NMR** (101 MHz, $CDCl_3$): δ 141.4 (CH, C-7), 141.0 (CH, C-7), 114.7 (CH_2, C-8), 114.4, (CH_2, C-8), 72.0 (CH, C-1* or C-2*), 69.2 (CH, C-1* or C-2*), 65.3 (CH, C-1* or C-2*), 62.8 (CH, C-1* or C-2*), 35.9 (CH_2, C-3), 35.7 (CH_2, C-3), 35.5 (CH, C-4), 32.72 (CH, C-4), 28.4 (CH_2, C-6), 27.5 (CH_2, C-6), 27.0 (CH_2, C-5), 25.1 (CH_2, C-5). *signals interchangeable. **EI-HRMS** m/z $[M+Na]^+$ calc. for $C_8H_{13}N_3O$ 190.0956, found 190.0952. **Achiral GC** 9.5 min for **5a** (method C) and 9.6 min for **5a′** (method C). **Chiral GC** 40.4, 40.4, 40.9 and 41.0 min (method A).

5a

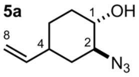

5a'

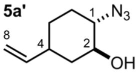

(1*S*,2*S,4R*)-2-cyano-1-methyl-4-(prop-1-en-2-yl)cyclohexan-1-ol (*trans*-**2b**)

zzFollowing the general procedure with *trans*-**2**, cyanide and HheG2 (OD_{600}=40, 44 h reaction time), cyano-alcohol *trans*-**2b** was obtained as a pure enantiomer as a colorless oil (11.5 mg, 23%, R_f = 0.31 in 3:1 pentane/EtOAc). **^{1}H NMR** (400 MHz, $CDCl_3$) δ 4.80–4.72 (2H, m, H-10), 2.80 (1H, m, H-2), 2.26 (1H, dddd, *J* = 15.3, 12.0, 6.7, 3.3, Hz, H-4), 1.96 (1H, ddd, *J* = 13.4, 12.3, 4.3 Hz, H-3), 1.88–1.74 (5H, m, H-3, H-6 and H-9), 1.72–1.64 (2H, m, H-6 and H-5), 1.60–1.54 (1H, m, H-5), 1.48 (3H, s, H-7). **^{13}C NMR** (101 MHz, $CDCl_3$): δ 148.0 (Cq, C-8), 120.8 (Cq, C-11), 109.7 (CH_2, C-10), 69.5 (Cq, C-1), 39.8 (CH, C-4), 39.1 (CH, C-2), 35.6 (CH_2, C-6), 29.5 (CH_3, C-7), 29.2 (CH_2, C-6), 26.0 (CH_2, C-3),

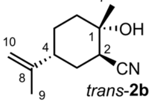

trans-**2b**

20.9 (CH_3, C-9). **EI-HRMS** m/z [M] calc. for $C_{11}H_{17}NO$ 179.13101, found 179.13102. **Achiral GC** 10.6 min (method D).

2-cyano-cyclopentan-1-ol (**3b**)

Following the general procedure with **3**, cyanide and HheG3 (OD_{600}=80, 46 h reaction time), cyano-alcohol **3b** was obtained as a mixture of enantiomers as a colorless oil (10.2 mg, 19%, R_f = 0.28 in 3:1 pentane/EtOAc).

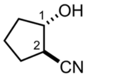

3b

^{1}H NMR (400 MHz, $CDCl_3$): δ (ppm) 4.49 (1H, ddd, *J* = 6.4, 5.1, 0.2 Hz, H-1), 2.80–2.63 (1H, m, H-2), 2.25–2.14 (1H, m, H-3), 2.11–2.00 (1H, m, H-5), 1.96–1.75 (3H, m, H-3 and H-4), 1.75–1.62 (1H, m, H-5). **^{13}C NMR** (101 MHz, $CDCl_3$): δ (ppm) 121.8 (Cq, C-6), 77.2 (CH, C-1), 37.6 (CH, C-2), 34.3 (CH_2, C-3), 28.9 (CH_2, C-5), 22.3 (CH_2, C-4). **EI-HRMS** m/z [M] calc. for C_6H_9NO 110.06059, found 151.05861. **Achiral GC** 4.2 min (method A). **Chiral GC** 38.5 and 38.7 min (method A).

2-cyano-1-methylcyclohexan-1-ol (**4b**)

Following the general procedure with *rac*-**4**, cyanide and HheG3 (OD_{600}=40, 46 h reaction time), cyano-alcohol **4b** was obtained as a mixture of enantiomers as a colorless oil (15.2 mg, 30%, R_f = 0.37 in 3:1 pentane/EtOAc). Although the other regioisomer (**4b′**) was produced during the reaction, NMR analysis only showed **4b**.

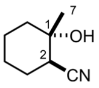

4b

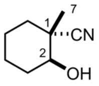

4b'

^{1}H NMR (400 MHz, $CDCl_3$) δ (ppm) 2.64 (1H, dd, *J* = 8.0, 4.1 Hz, H-2), 2.08–1.96 (1H, m, H-3), 1.79–1.63 (4H, m, H-3, H-6, and H-4/5), 1.54–1.38 (3H, m, H-4 and H-5), 1.42 (3H, s, H-7). **^{13}C NMR** (101 MHz, $CDCl_3$) δ (ppm) 120.93 (Cq, C-8), 70.49 (Cq, C-1), 40.69 (CH, C-2), 37.74 (CH_2, C-6), 26.57 (CH_2, C-4*), 26.01 (CH_3, C-7), 23.12 (CH_2, C-5*), 22.12 (CH_2, C-3*). *signals interchangeable. **EI-HRMS** m/z [M] calc. for $C_8H_{13}NO$ 139.09971, found 139.09848. **Achiral GC** 5.9 min for **4b** (method A) and 6.5 min for **4b′** (method A). **Chiral GC** 38.3 and 38.8 min (for enantiomers of **4b**, method A).

2-cyano-4-vinylcyclohexan-1-ol (**5b**) and 1-cyano-4-vinylcyclohexan-2-ol (**5b′**)

Following the general procedure with *rac*-**5**, cyanide and HheG2 (OD_{600}=40, 44 h reaction time), cyano-alcohols **5b** and **5b′** were obtained as a mixture of regioisomers as a colorless oil (11.9 mg, 24%, R_f = 0.28 in 3:1 pentane/EtOAc).

5b: **^{1}H NMR** (400 MHz, $CDCl_3$): δ (ppm) 5.76 (1H, dddd, *J* = 15.8, 10.8, 6.1, 3.8 Hz, H-7), 5.13–4.97 (2H, m, H-8), 4.02 (1H, m, H-1), 2.83 (1H, m, H-2), 2.48–2.40 (1H, m, H-4), 1.96–1.90 (2H, m, H-3), 1.89–1.82 (2H, m, H-6), 1.64–1.61 (2H, m, H-5). **^{13}C NMR** (101 MHz, $CDCl_3$) δ (ppm) 140.82 (CH, C-7), 120.93 (Cq, C-9), 114.77 (CH_2, C-8), 68.18 (CH, C-1), 35.92 (CH, C-4), 33.47 (CH_2, C-6), 29.69 (CH_2, C-3), 29.34 (CH_2, C-5), 26.11 (CH, C-2).

5b

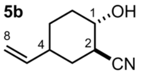

5b'

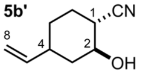

5b′: **^{1}H NMR** (400 MHz, $CDCl_3$): δ (ppm) 5.76 (1H, dddd, *J* = 15.8, 10.8, 6.1, 3.8 Hz, H-7), 5.13–4.97 (2H, m, H-8), 4.17 (1H, m, H-2), 2.73 (1H, m, H-1), 2.57–2.48 (1H, m, H-4), 2.07–2.03 (1H, m, H-6), 1.89–1.82 (1H, m, H-6), 1.83–1.79 (1H, m, H-3), 1.77–1.72 (1H, m, H-3), 1.67 (1H, m, H-5), 1.61–1.58 (1H, m, H-5). **^{13}C NMR** (101 MHz, $CDCl_3$) δ (ppm) 141.64 (CH, C-7), 120.71 (Cq, C-9), 114.18 (CH_2, C-8), 66.96 (CH, C-2), 36.25 (CH_2, C-3), 34.90 (CH, C-4), 34.68 (CH_2, C-5), 27.95 (CH_2, C-1), 23.07 (CH_2, C-6).

EI-HRMS m/z [M] calc. for $C_9H_{13}NO$ 151.09971, found 151.09863. **Achiral GC** 9.0 min for **5b** and 9.1 for **5b′** (method A). **Chiral GC** 42.2, 43.6, 43.7, 43.9, 45.9 and 45.9 min (method A).

hexahydrobenzo[d]oxazol-2(3H)-one (**1c**)

Following the general procedure with **1**, cyanate and HheG3 (OD_{600}=40, 20 h reaction time), oxazolidinone **1c** was obtained as a mixture of enantiomers as a colorless solid (33.4 mg, 67%, filtered using EtOAc).

1c

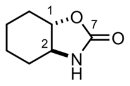

^{1}H NMR (400 MHz, DMSO-d6) δ (ppm) 7.56 (1H, bs, N-H), 3.75 (1H, ddd, *J* = 11.7, 10.9, 3.7 Hz, H-1), 3.18 (1H, ddd, *J* = 11.0, 3.6, 0. Hz, H-2), 2.05–1.96 (1H, m, H-3), 1.94–1.86 (1H, m, H-6), 1.78 (1H, m, H-4), 1.71–1.63 (1H, m, H-5), 1.55 (1H, ddd, *J* = 23.0, 11.9, 4.0 Hz, H-3), 1.38–1.23 (3H, m, H-6 , H-4, and H-5). **^{13}C NMR** (101 MHz, DMSO-d6): δ (ppm) 159.73 (Cq, C-7), 82.42 (CH, C-1), 59.79 (CH, C-2), 28.66 (CH_2, C-3), 28.01 (CH_2, C-6), 23.24 (CH_2, C-4), 23.03 (CH_2, C-5). **EI-HRMS** m/z [M] calc. for $C_7H_{11}NO_2$ 141.07898, found 141.07776. **Achiral GC** 10.1 min (method A). **Chiral GC** 49.2 and 37.6 min (method A).

(3a*S*,5*R*,7a*S*)-7a-methyl-5-(prop-1-en-2-yl)hexahydrobenzo[d]oxazol-2(3H)-one (*trans*-**2c**)

Following the general procedure with *trans*-**2**, cyanate and HheG3 (OD_{600}=40, 17 h reaction time), oxazolidinone *trans*-**2c** was obtained as a pure enantiomer as a colorless solid (30.4 mg, 61%, R_f = 0.28 in 3:1 pentane/EtOAc).

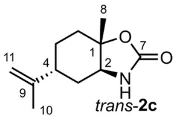

trans-**2c**

^{1}H NMR (400 MHz, $CDCl_3$) δ (ppm) 4.95 (1H, m, H-11), 4.88 (1H, dt, *J* = 1.8, 0.9 Hz, H-11), 3.60 (1H, ddd, *J* = 13.3, 3.5, 0.9 Hz, H-2), 2.52–2.45 (1H, m, H-4), 2.16–2.08 (1H, m, H-3), 2.05–1.99 (2H, m, H-6 and H-3), 1.89 (1H, dddd, *J* = 12.0, 5.5, 2.5, 0.9 Hz, H-6), 1.80–1.71 (5H, m, H-5 and H-10), 1.39 (3H, s, H-8). **^{13}C NMR** (101 MHz, $CDCl_3$) δ (ppm) 160.89 (Cq, C-7), 146.80 (CH_2, C-9), 111.66 (CH_2, C-11), 85.49 (Cq, C-1), 58.30 (CH, C-2), 38.32 (CH, C-4), 32.63 (CH_2, C-6), 26.46 (CH_2, C-3), 25.55 (CH_2, C-5), 22.80 (CH_3, C-8), 17.02 (CH_3, C-10). **EI-HRMS** m/z [M] calc. for $C_{11}H_{17}NO_2$ 195.12593, found 195.12836. **Achiral GC** 9.0 min (method D).

7a-methylhexahydrobenzo[d]oxazol-2(3H)-one (**4c**) and

3a-methylhexa-hydrobenzo[d]oxazol-2(3H)-one (**4c′**)

Following the general procedure with *rac*-**4**, cyanate and HheG3 (OD_{600}=40, 17 h reaction time), oxazolidinones **4c** and **4c′** were obtained as a mixture of isomers as a colorless solid (19.3 mg, 39%, R_f = 0.48 in 1:1 pentane/EtOAc).

4c

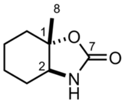

4c'

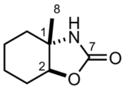

4c: **^{1}H NMR** (400 MHz, $CDCl_3$) δ (ppm) 3.43 (1H, ddd, *J* = 12.7, 3.5, 0.9 Hz, H-2), 2.04–1.38 (8H, m, H-3, H-4, H-5, and H-6), 1.34 (3H, s, H-8). **^{13}C NMR** (101 MHz, $CDCl_3$) δ (ppm) 161.01 (Cq, C-7), 84.97 (Cq, C-1), 63.21 (CH, C-2), 34.77 (CH_2, C-6), 24.88*, 23.73*, 22.75*, 16.95 (CH_3, C-8). **Achiral GC** 10.3 min (method A). **Chiral GC** 49.8 and 50.1 min (method A).

4c′: **^{1}H NMR** (400 MHz, $CDCl_3$) δ (ppm) 3.96 (1H, ddd, *J* = 12.9, 3.6, 0.8 Hz, H-2), 2.04–1.38 (8H, m, H-3, H-4, H-5, and H-6), 1.20 (3H, s, H-8). **^{13}C NMR** (101 MHz, $CDCl_3$) δ (ppm) 160.72 (Cq, C-7), 85.26 (CH, C-2), 59.95 (Cq, C-1), 34.72 (CH_2, C-6), 23.86*, 23.67*, 21.63*, 18.36 (CH_3, C-8). *signals interchangeable. **EI-HRMS** m/z [M] calc. for $C_8H_{13}NO_2$ 155.09463, found 155.09664. **Achiral GC** 10.2 min (method A). **Chiral GC** 48.4 and 48.7 min (method A).

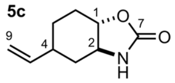

5-vinylhexahydrobenzo[d]oxazol-2(3H)-one (**5c**)

Following the general procedure with *rac*-**5**, cyanate and HheG3 (OD_{600}=40, 32 h reaction time), oxazolidinone **5c** was obtained as a mixture of enantio- and diastereomers as a colorless oil (6.5 mg, 13%, R_f = 0.31 in 2:3 pentane/EtOAc).

^{1}H NMR (400 MHz, $CDCl_3$) δ (ppm) 5.84 (1H, ddd, *J* = 17.3, 10.7, 5.3 Hz, H-8), 5.16–5.07 (2H, m, H-9), 3.96–3.85 (1H, ddd, *J* = 11.9, 11.1, 3.6 H, H-1), 3.51 (1H, dddd, *J* = 12.2, 11.0, 3.6, 1.1 Hz, H-2), 2.68 (1H, m, H-4), 2.14–1.98 (3H, m, H-3, H-5 and H-6), 1.84 (1H, m, H-6), 1.77–1.65 (2H, m, H-3 and H-5). **^{13}C NMR** (101 MHz, $CDCl_3$) δ (ppm) 160.78 (Cq, C-7), 140.64 (CH, C-8), 115.22 (CH_2, C-9), 84.20 (CH, C-1), 56.66 (CH, C-2), 35.37 (CH, C-4), 33.80 (*C*H_2, C-3), 27.22 (CH_2, C-5), 25.11 (CH_2, C-6). **EI-HRMS** m/z [M] calc. for $C_9H_{13}NO_2$ 167.09463, found 167.09598. **Achiral GC** 10.83 min (method A).

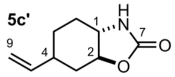

6-vinylhexahydrobenzo[d]oxazol-2(3H)-one (**5c′**)

Following the general procedure with *rac*-**5**, cyanate and HheG3 (OD_{600}=40, 32 h reaction time), oxazolidinone **5c′** was obtained as a mixture of enantio- and diastereomers as a colorless oil (22.2 mg, 44%, R_f = 0.58 in 2:3 pentane/EtOAc).

^{1}H NMR (400 MHz, $CDCl_3$) δ (ppm) 5.86 (1H, ddd, *J* = 17.4, 10.7, 5.2 Hz, H-8), 5.17–5.09 (2H, m, H-9), 4.07 (1H, ddd, *J* = 12.5, 11.0, 3.7 Hz, H-2), 3.40–3.29 (1H, m, H-1), 2.79 (1H, m, H-4), 2.31–2.21 (1H, m, H-3), 2.00–1.85 (3H, m, H-3, H-5, and H-6), 1.73–1.60 (2H, m, H-5 and H-6). **^{13}C NMR** (101 MHz, $CDCl_3$) δ (ppm) 160.59 (Cq, C-7), 140.56 (CH, C-8), 115.29 (CH_2, C-9), 80.32 (CH, C-2), 61.41 (CH, C-1), 35.96 (CH, C-4), 32.85 (CH_2, C-3), 27.30 (CH_2, C-6), 25.78 (CH_2, C-5). **EI-HRMS** m/z [M] calc. for $C_9H_{13}NO_2$ 167.09463, found 167.09608. **Achiral GC** 10.85 min (method A).

Chiral GC for **5c** and **5c′** 51.6, 52.2, 52.3 and 52.4 min (method A).

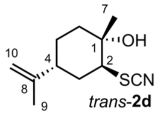

(1*S*,2*S*,4*R*)-1-methyl-4-(prop-1-en-2-yl)-2-thiocyanatocyclohexan-1-ol (*trans*-**2d**)

Following the general procedure with *trans*-**2**, thiocyanate and HheG3 (OD_{600}=40, 46 h reaction time), thiocyanato-alcohol *trans*-**2d** was obtained as pure enantiomer as a colorless solid (6.8 mg, 14%, R_f = 0.31 in 7:1 pentane/EtOAc). **^{1}H NMR** (400 MHz, $CDCl_3$) δ (ppm) 4.79 (2H, m, H-10), 3.60 (1H, m, H-2), 2.33 (1H, ddd, *J* = 14.1, 10.5, 3.6 Hz, H-3), 2.22–2.16 (1H, m, H-4), 2.02 (1H, dddd, *J* = 14.1, 4.9, 3.4, 1.3 Hz, H-3), 1.80–1.64 (7H, m, H-5, H-6, and H-9), 1.41 (3H, s, H-7). **^{13}C NMR** (101 MHz, $CDCl_3$) δ (ppm) 147.34 (Cq, C-8), 112.33 (Cq, C-11), 110.40 (CH_2, C-10), 71.78 (Cq, C-1), 56.86 (CH, C-2), 38.56 (CH, C-4), 34.73 (CH_2, C-6), 32.99 (CH_2, C-3), 27.88 (CH_3, C-7), 26.01 (CH_2, C-5), 21.36 (*C*H_3, C-9). **EI-HRMS** m/z [M] calc. for $C_{11}H_{17}NOS$ 211.10308, found 211.10287. **Achiral GC** 12.4 min (method D).

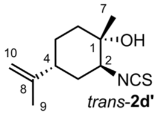

trans-**2d'**

(1*S*,2*S*,4*R*)-1-methyl-4-(prop-1-en-2-yl)-2-isothiocyanatocyclohexan-1-ol (*trans*-**2d′**)

Following the general procedure with *trans*-**2**, thiocyanate and HheG3 (OD_{600}=40, 46 h reaction time), isothiocyanato-alcohol *trans*-**2d′** was obtained as pure enantiomer as a colorless solid (9.8 mg, 20%, R_f = 0.41 in 7:1 pentane/EtOAc). **^{1}H NMR** (400 MHz, $CDCl_3$): δ (ppm) 4.78−4.73 (2H, m, H-10), 3.72 (1H, m, H-2), 2.21 (1H, m, H-4), 1.98 (1H, ddd, *J* = 13.6, 12.1, 3.3 Hz, H-3), 1.87 (1H, m, *J* = 13.7, 3.5, 1.9 Hz, H-3), 1.78–1.53 (7H, m, H-5, H-6, and H-9), 1.37 (3H, s, H-7). **^{13}C NMR** (101 MHz, $CDCl_3$) δ (ppm) 148.36 (Cq, C-8), 109.78 (CH_2, C-10), 70.83 (Cq, C-1), 62.53 (CH, C-2), 38.33 (CH, C-4), 34.23 (CH_2, C-6), 32.67 (CH_2, C-3), 28.41 (CH_3, C-7), 26.03 (CH_2, C-5), 21.16 (CH_3, C-9). **EI-HRMS** m/z [M] calc. for $C_{11}H_{17}NOS$ 211.10308, found 211.10224. **Achiral GC** 12.9 min (method D).

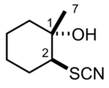

4d

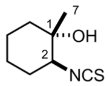

4d'

1-methyl-2-thiocyanatocyclohexan-1-ol (**4d**) and 2-isothiocyanato-1-methylcyclohexan-1-ol (**4d′**)

Following the general procedure with *rac*-**4**, thiocyanate and HheG3 (OD_{600}=40, 48 h reaction time), thiocyanato-alcohol **4d** and isothiocyanato-alcohol **4d′** were obtained as a mixture of inseparable isomers as a colorless solid (9.0 mg, 18%, R_f = 0.65 in 1:1 pentane/EtOAc). NMR data is given as available, as the remaining signals showed complete overlap and could not be distinguished

4d: ^{1}H NMR (400 MHz, $CDCl_3$) δ (ppm) 3.28 (1H, dd, *J* = 11.9, 4.0 Hz, CH-SCN). **Achiral GC** 9.8 min for both regioisomers (method A). **Chiral GC** 45.4 and 45.5 min for **4d** (method A).

4d′: **^{1}H NMR** (400 MHz, $CDCl_3$) δ (ppm) 3.53 (1H, dd, *J* = 12.9, 3.5 Hz, CH-NCS).

EI-HRMS m/z [M] calc. for $C_8H_{13}NOS$ 171.07178, found 171.07169. **Achiral GC** 9.4 and 9.5 min (method A). **Chiral GC** 42.4 and 42.9 min for **4d′** (method A).

2-thiocyanato-4-vinylcyclohexan-1-ol (**5d**), 2-isothiocyanato-4-vinylcyclohexan-1-ol (**5d′**), 1-thiocyanato-4-vinylcyclohexan-2-ol (**5d″**), 1-isothiocyanato-4-vinylcyclohexan-2-ol (**5d‴**)

Following the general procedure with *rac*-**5**, thiocyanate and HheG3 (OD_{600}=40, 48 h reaction time), thiocyanato-alcohols **5d, 5d″** and isothiocyanato-alcohols **5d′**, **5d‴** were obtained as a mixture of isomers as a colorless oil (14.8 mg, 30%, R_f = 0.73 in 1:2 pentane/EtOAc). NMR data is given as available, as the remaining signals showed complete overlap and could not be distinguished. For this product, extensive 2D-NMR analysis allowed an assignment of most relevant signals for all isomers.

5d

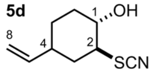

5d: **^{1}H NMR** (500 MHz, $CDCl_3$) δ (ppm) 5.92–5.74 (1H, m, H-7), 5.22–5.03 (2H, m, H-8), 3.75−3.67 (1H, m, H-1), 3.26−3.20 (1H, m, H-2), 2.60−2.54 (1H, m, H-4), 2.29−2.26 (1H, m, H-3). **^{13}C NMR** (126 MHz, $CDCl_3$) δ (ppm) 72.11 (CH, C-1), 51.55 (CH, C-2), 36.42 (CH, C-4), 35.39 (CH_2, C-3). **Achiral GC** 10.3 min (method A).

5d'

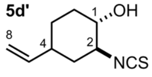

5d′: **^{1}H NMR** (500 MHz, $CDCl_3$) δ (ppm) 5.92–5.74 (1H, m, H-7), 5.22–5.03 (2H, m, H-8), 3.66−3.59 (1H, m, H-1), 2.74−2.69 (1H, m, H-4). **^{13}C NMR** (126 MHz, $CDCl_3$) δ (ppm) 35.25 (CH, C-4). **Achiral GC** 10.7 min (method A).

5d''

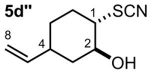

5d″: **^{1}H NMR** (500 MHz, $CDCl_3$) δ (ppm) 5.92–5.74 (1H, m, H-7), 5.22–5.03 (2H, m, H-8), 3.95−3.89 (1H, m, H-2), 3.17−3.10 (1H, m, H-1), 2.67−2.60 (1H, m, H-4), 2.11−2.09 (1H, m, H-3). **^{13}C NMR** (126 MHz, $CDCl_3$) δ (ppm) 68.92 (CH, C-2), 54.30 (CH, C-1), 37.40 (CH_2, C-3), 35.83 (CH, C-4). **Achiral GC** 10.4 min (method A).

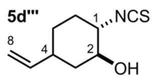

5d‴: **^{1}H NMR** (500 MHz, $CDCl_3$) δ (ppm) 5.92–5.74 (1H, m, H-7), 5.22–5.03 (2H, m, H-8), 4.28−4.20 (1H, m, H-2), 3.48−3.41 (1H, m, H-1), 2.85−2.80 (1H, m, H-4), 1.99−1.97 (1H, m, H-3). **^{13}C NMR** (126 MHz, $CDCl_3$) δ (ppm) 85.35 (CH, H-2), 63.66 (CH, C-1), 35.86 (CH, C-4), 32.73 (CH_2, C-3). **Achiral GC** 10.7 min (method A).
EI-HRMS m/z [M] calc. for $C_9H_{13}NOS$ 183.07178, found 183.0709. **Chiral GC** for **5d**, **5d′**, **5d″** and **5d‴** 46.6, 46.6, 46.9, 47.0, 47.3, 47.5, 47.6, 47.8, 48.0, 48.1 and 48.5 min (method A).

1-hydroxy-4-vinylcyclohexyl formate (**5e**) and 2-hydroxy-4-vinylcyclohexyl formate (**5e′**)
Following the general procedure with **5**, formate and HheG3 (OD_{600}=80, 20 h reaction time), formates **5e** and **5e′** were obtained as a mixture of isomers as a colorless solid (5.1 mg, 10%, R_f = 0.31 in 5:1 pentane/EtOAc). NMR data is given as available, as the remaining signals showed complete overlap and could not be distinguished. NMR data are listed as the sum of both isomers.

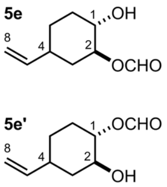

^{1}H NMR (400 MHz, $CDCl_3$) δ (ppm) 8.12−8.09 (2H, m, H-9), 5.81 (2H, ddd, *J* = 17.4, 10.5, 5.9 Hz, H-7), 5.16–5.07 and 5.06–5.02 (4H, m, H-8), 5.01−4.94 and 4.86−4.80 (2H, m, H-1/2), 3.94−3.88 and 3.79−3.73 (2H, m, H-1/2), 2.58–2.43 (2H, m, H-4), 2.03–1.84 (6H, m, H-3, H-6 and H-5), 1.72–1.59 (6H, m, H-3, H-6 and H-5). **^{13}C NMR** (101 MHz, $CDCl_3$) δ (ppm) 160.83 (CH, C-9), 141.86 and 141.41 (CH, C-7), 114.38 and 114.05 (*C*H_2, C-8), 75.09 and 73.66 (*C*H, C-1/2), 69.88 and 67.91 (CH, C-1/2), 35.83 (CH, C-4), 35.54 (CH_2, C-3), 35.12 (CH, C-4), 32.07 (CH_2, C-3), 28.42 (CH_2, C-6*), 26.99 (CH_2, C-6*), 26.70 (CH_2, C-5*), 25.24 (CH_2, C-5*). *signals interchangeable. **EI-HRMS** m/z [M] calc. for $C_9H_{14}O_3$ 170.09429, found 170.09198. **Achiral GC** 8.6 min for **5e** and 8.7 min for **5e′** (method A). **Chiral GC** 40.2, 41.1, 41.2 and 41.7 min (method A).

2-nitrocyclohexan-1-ol (**1f**)
Following the general procedure with **1**, nitrite and HheG3 (OD_{600}=80, 17 h reaction time), nitro-alcohol **1f** was obtained as a mixture of enantiomers as a colorless oil (4.3 mg, 9%, R_f = 0.20 in 7:1 pentane/EtOAc).

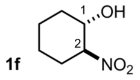

^{1}H NMR (400 MHz, $CDCl_3$) δ (ppm) 4.28 (1H, ddd, *J* = 12.0, 9.5, 4.3 Hz, H-2), 4.09 (1H, ddd, *J* = 10.8, 9.5, 4.9 Hz, H-1), 2.38–1.29 (8H, m, H-3, H-4, H-5, and H-6). **^{13}C NMR** (101 MHz, $CDCl_3$) δ (ppm) 91.43 (CH, C-2), 71.21 (CH, C-1), 32.99 (CH_2, C-6), 30.58 (CH_2, C-4), 24.13* (CH_2, C-5), 23.72* (CH_2, C-3). *signals interchangeable. **EI-HRMS** m/z [M-H_2O] calc. for $C_6H_{11}NO_2$ 127.06333, found 127.06322. **Achiral GC** 5.6, 6.4 and 7.1 min (method A). **Chiral GC** 40.9 and 41.5 min (method A).

(+)-*trans*-(1*S*,2*S*)-1-methyl-2-nitro-4-(prop-1-en-2-yl)-cyclohexan-1-ol (*trans*-**2f**)

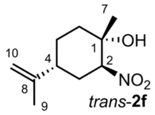

trans-**2f**

Following the general procedure with *trans*-**2**, nitrite and HheG3 (OD_{600}=40, 19 h reaction time), nitro-alcohol *trans*-**2f** was obtained as a yellowish solid (6.9 mg, 9%, R_f = 0.22 in 10:1 pentane/EtOAc).

^{1}H NMR (400 MHz, $CDCl_3$) δ (ppm) 4.86–4.74 (2H, m, H-10), 4.63 (1H, ddd, *J* = 5.5, 4.8, 0.9 Hz, H-2), 2.54 (1H, m, H-4), 2.21 (1H, ddd, *J* = 14.4, 9.6, 4.7 Hz, H-3), 2.14–2.03 (2H, m, H-3 and H-6), 1.77–1.66 (6H, m, H-5, H-6 and H-9), 1.31 (3H, s, H-7). **^{13}C NMR** (101 MHz, $CDCl_3$) δ (ppm) 147.03 (Cq, C-8), 110.55 (CH_2, C-10), 90.10 (CH, H-2), 70.20 (Cq, C-1), 37.65 (CH, C-4), 34.61 (CH_2, C-6), 30.66 (CH_2, C-3), 25.60 (CH_2, C-5), 25.33 (CH_3, C-7), 21.42 (CH_3, C-9). **EI-HRMS** m/z [M] calc. for $C_{10}H_{17}NO_3$ 199.12084, found 199.12195. **Achiral GC** 10.6 min (method D).

NMR spectra

All NMR data for the compounds detailed above are freely available for download at zenodo.org.[8]

Achiral and chiral GC chromatograms

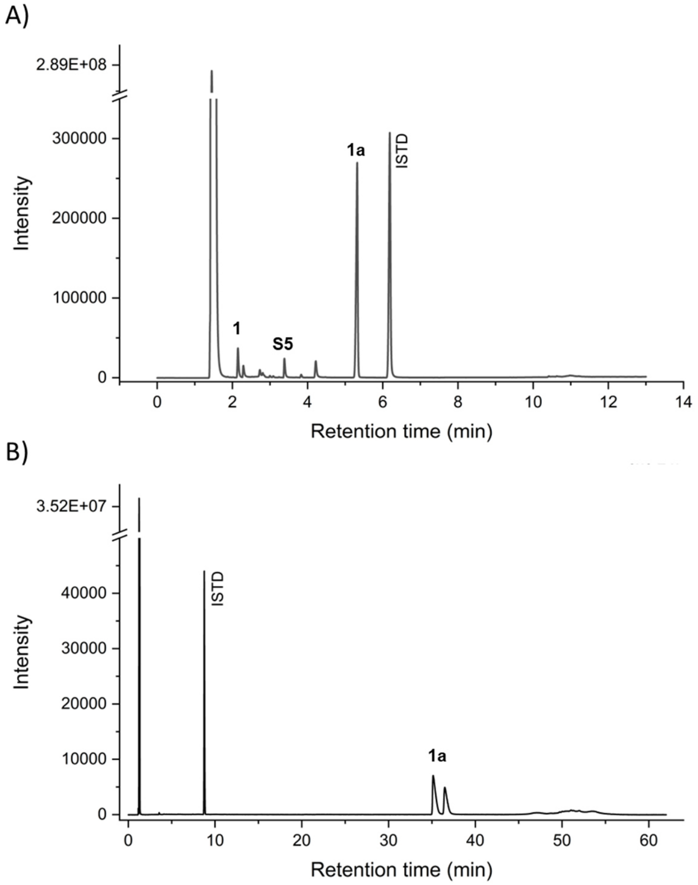

Figure S3: Achiral (A) and chiral (B) GC-chromatogram of the reaction using purified HheG3, cyclohexene oxide (**1**) as substrate and azide as nucleophile after 2 h. Measured with achiral method A and chiral method B.

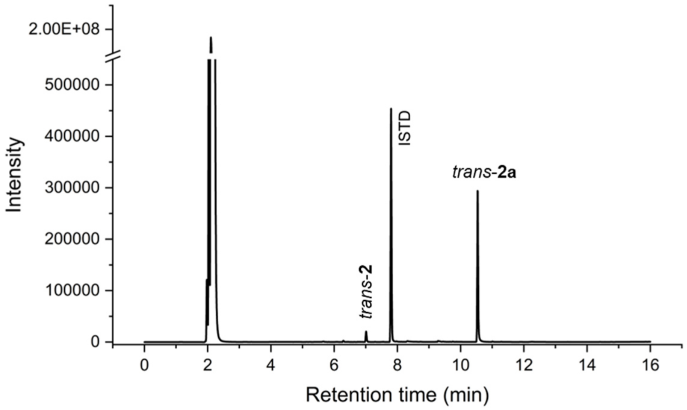

Figure S4: Achiral GC-chromatogram of the reaction using purified HheG3, (+)-*trans*-limonene oxide (**2**) as substrate and azide as nucleophile after 2 h. Measured with achiral method D.

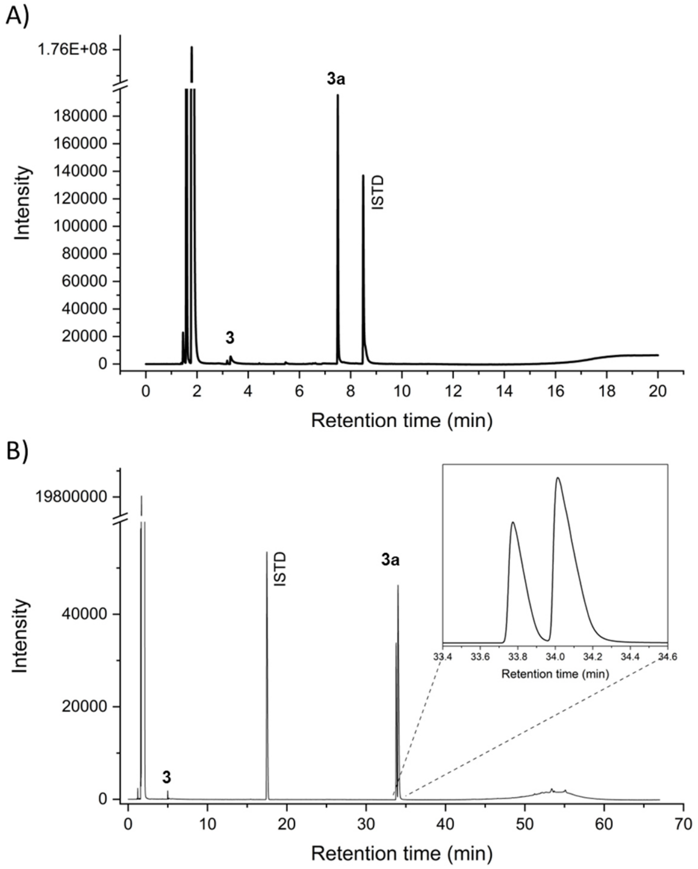

Figure S5: Achiral (A) and chiral (B) GC-chromatogram of the reaction using purified HheG3, cyclopentene oxide (**3**) as substrate and azide as nucleophile after 24 h. Measured with achiral method C and chiral method A.

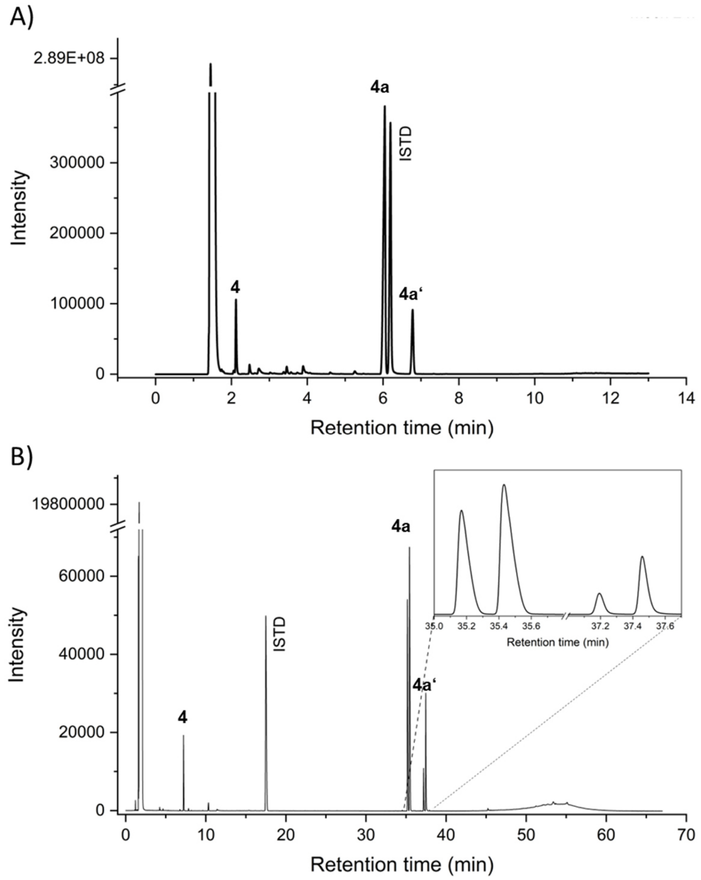

Figure S6: Achiral (A) and chiral (B) GC-chromatogram of the reaction using purified HheG3, methyl-epoxy-cyclohexane (**4**) as substrate and azide as nucleophile after 2 h. Measured with achiral method A and chiral method A.

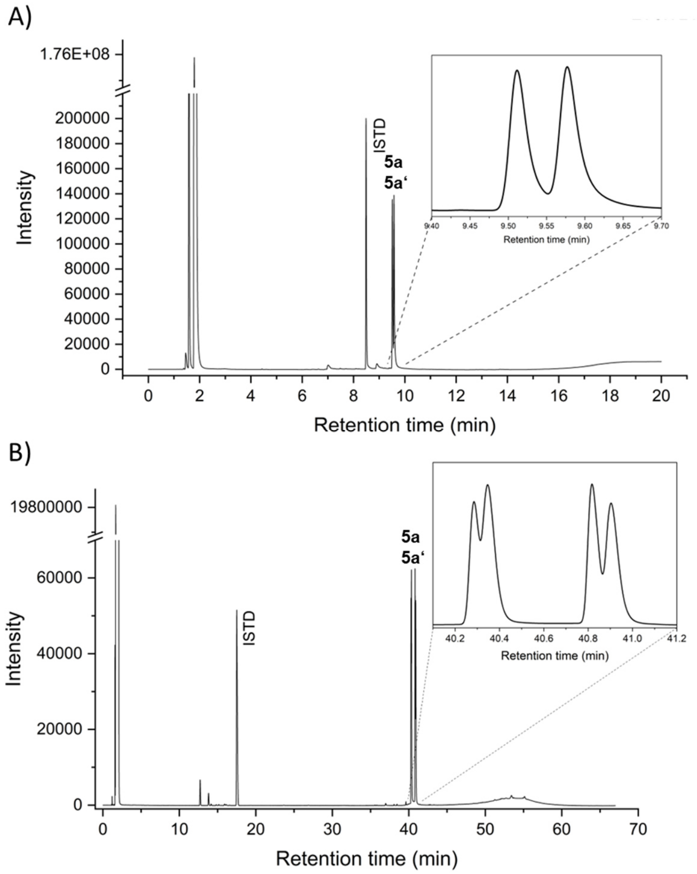

Figure S7: Achiral (A) and chiral (B) GC-chromatogram of the reaction using purified HheG3, epoxy-vinyl-cyclohexane (**5**) as substrate and azide as nucleophile after 2 h. Measured with achiral method C and chiral method A.

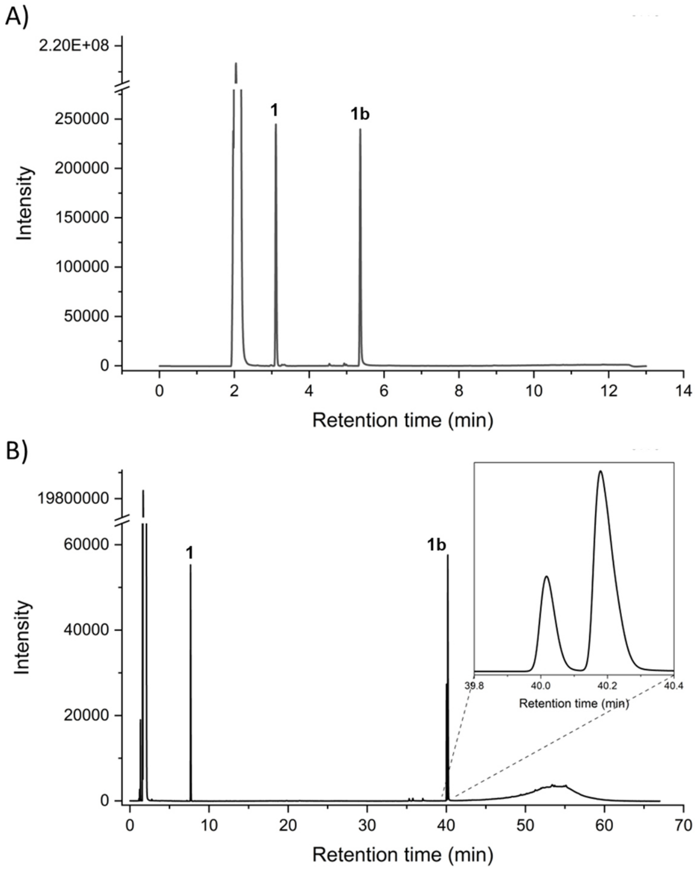

Figure S8: Achiral (A) and chiral (B) GC-chromatogram of the reaction using purified HheG3, cyclohexene oxide (**1**) as substrate and cyanide as nucleophile after 24 h. Measured with achiral method B and chiral method A.

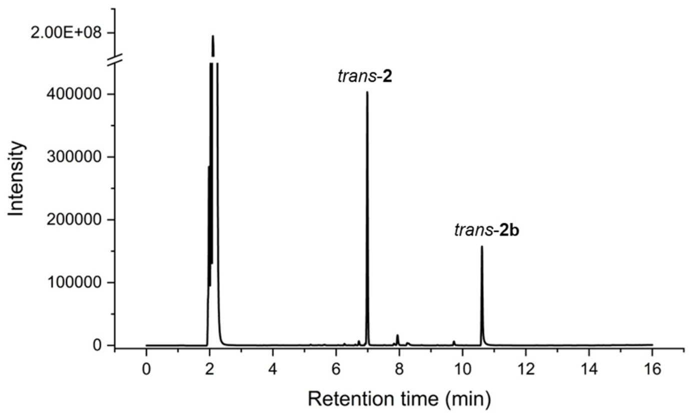

Figure S9: Achiral GC-chromatogram of the reaction using purified HheG3, (+)-*trans*-limonene oxide (*trans*-**2**) as substrate and cyanide as nucleophile after 24 h. Measured with achiral method D.

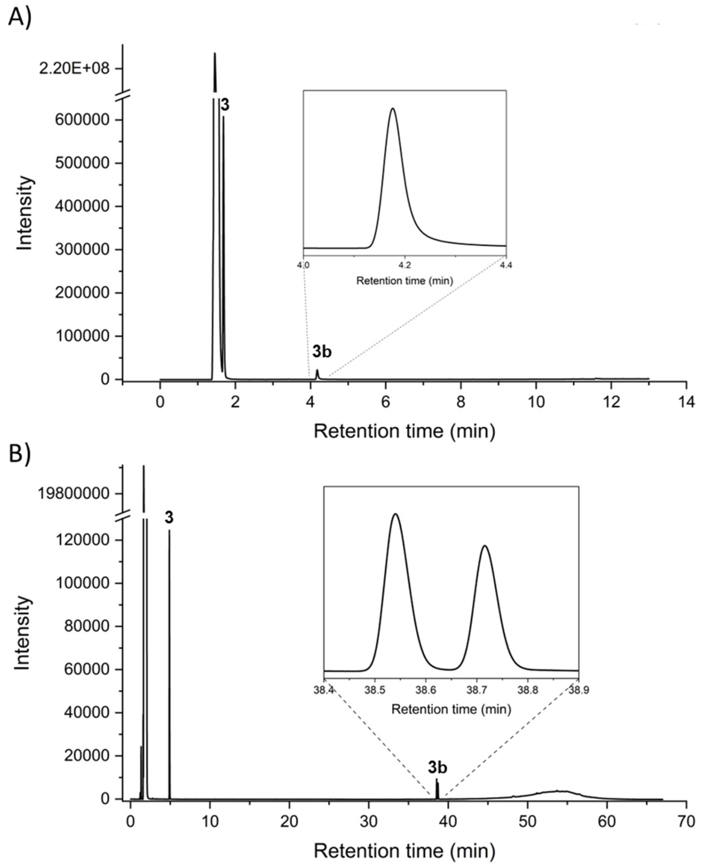

Figure S10: Achiral (A) and chiral (B) GC-chromatogram of the reaction using purified HheG3, cyclopentene oxide (**3**) as substrate and cyanide as nucleophile after 24 h. Measured with achiral and chiral method A.

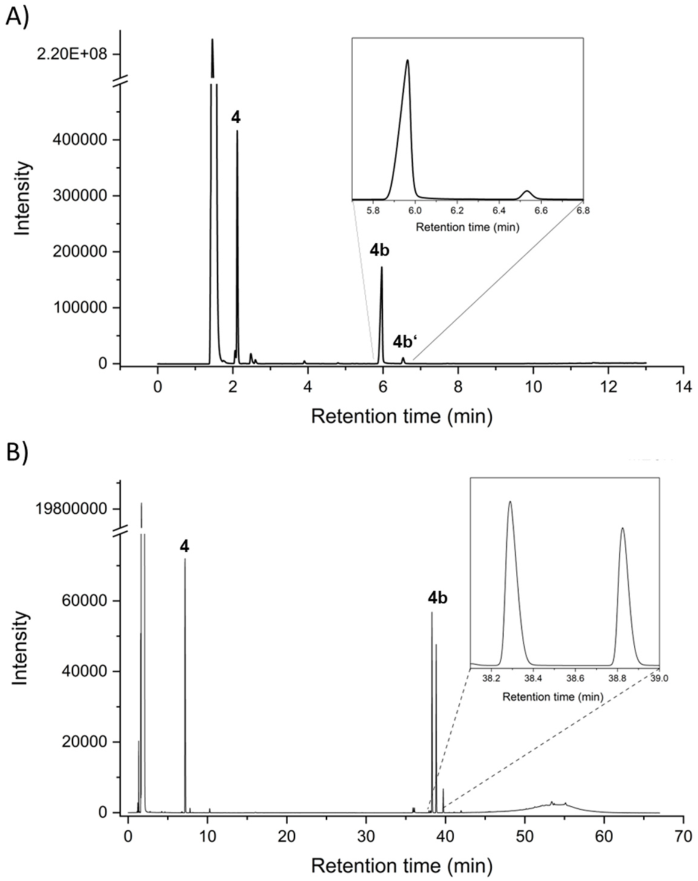

Figure S11: Achiral (A) and chiral (B) GC-chromatogram of the reaction using purified HheG3, methyl-epoxy-cyclohexane (**4**) as substrate and cyanide as nucleophile after 24 h. Measured with achiral and chiral method A.

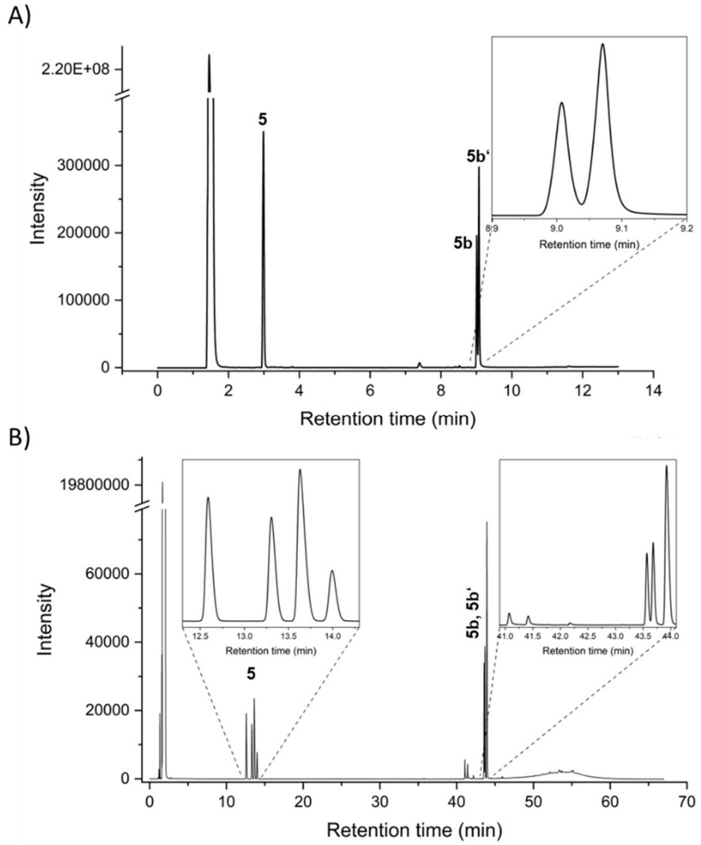

Figure S12: Achiral (A) and chiral (B) GC-chromatogram of the reaction using purified HheG3, epoxy-vinyl-cyclohexane (**5**) as substrate and cyanide as nucleophile after 24 h. Measured with achiral and chiral method A.

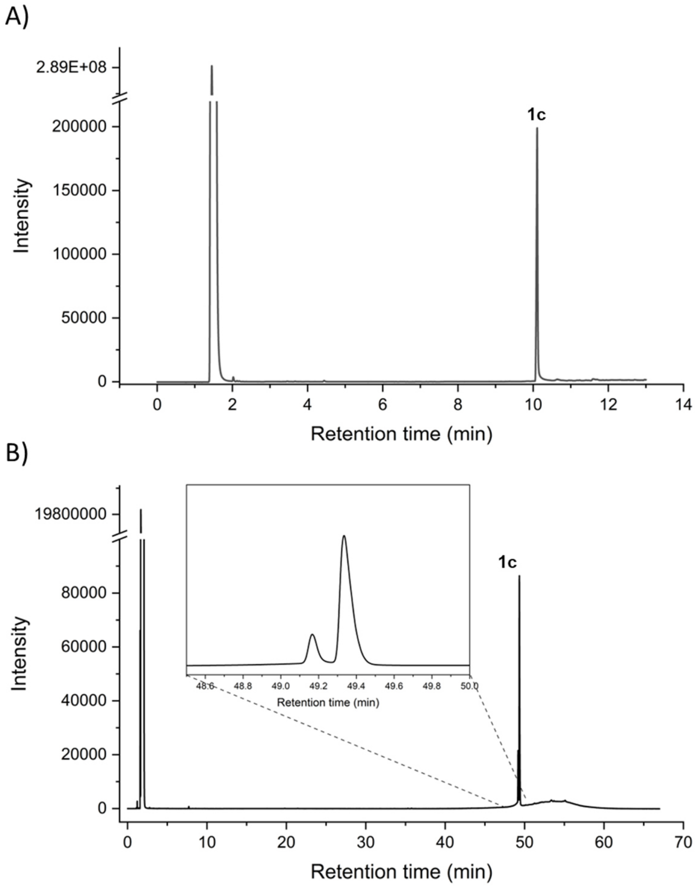

Figure S13: Achiral (A) and chiral (B) GC-chromatogram of the reaction using purified HheG3, cyclohexene oxide (**1**) as substrate and cyanate as nucleophile after 24 h. Measured with achiral and chiral method A.

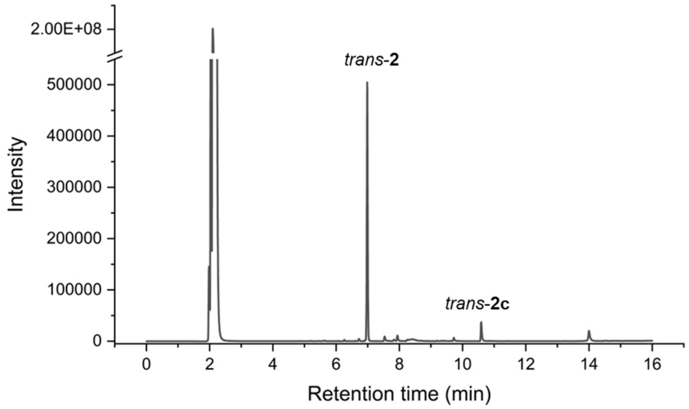

Figure S14: Achiral GC-chromatogram of the reaction using purified HheG3, (+)-*trans*-limonene oxide (**2**) as substrate and cyanate as nucleophile after 24 h. Measured with achiral method D.

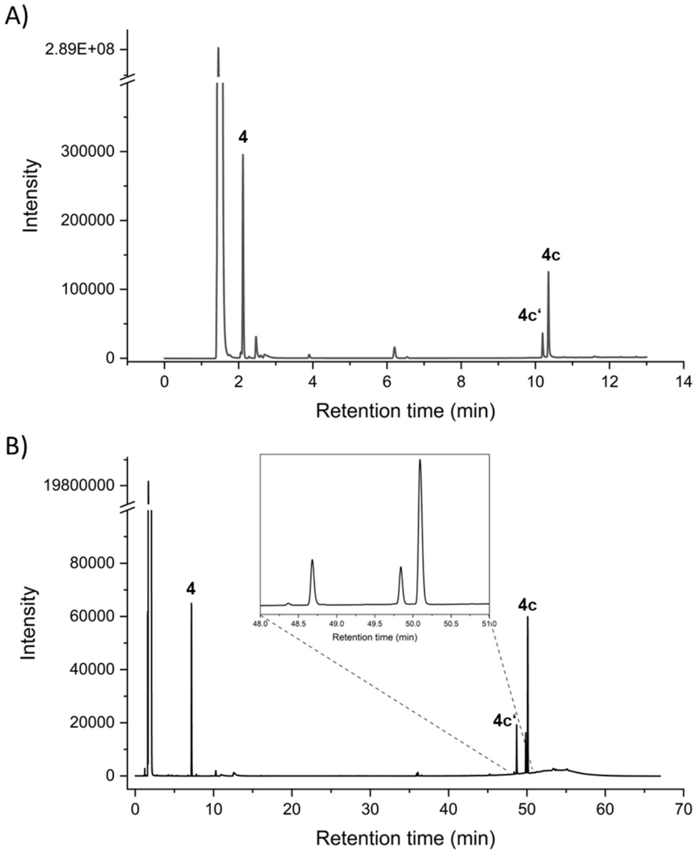

Figure S15: Achiral (A) and chiral (B) GC-chromatogram of the reaction using purified HheG3, methyl-epoxy-cyclohexane (**4**) as substrate and cyanate as nucleophile after 24 h. Measured with achiral and chiral method A.

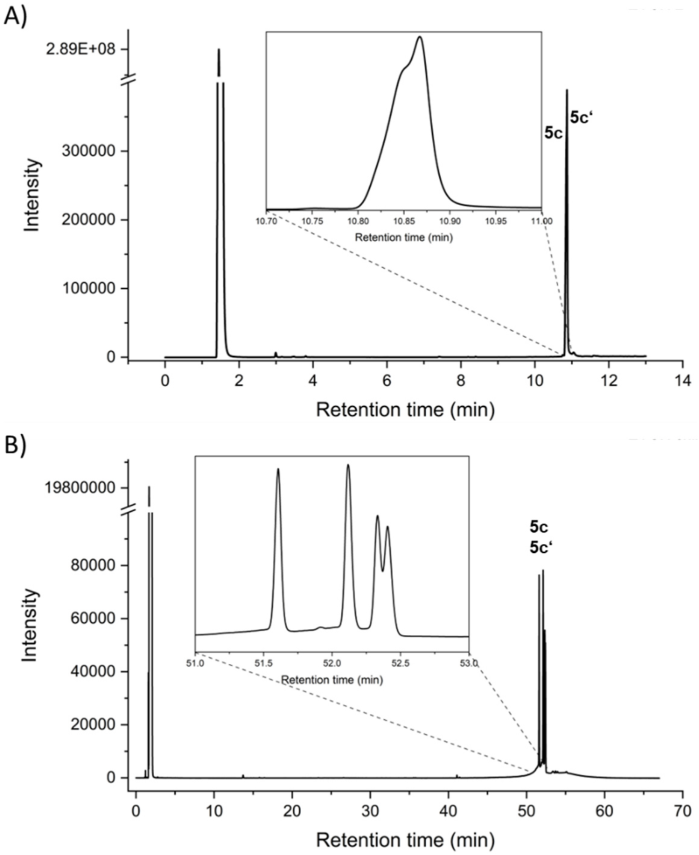

Figure S16: Achiral (A) and chiral (B) GC-chromatogram of the reaction using purified HheG3, epoxy-vinyl-cyclohexane (**5**) as substrate and cyanate as nucleophile after 24 h. Measured with achiral and chiral method A.

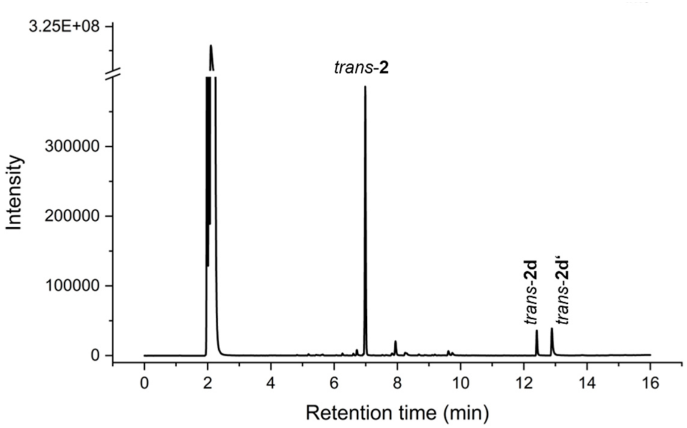

Figure S17: Achiral GC-chromatogram of the reaction using purified HheG3, (+)-*trans*-limonene oxide (**2**) as substrate and thiocyanate as nucleophile after 24 h. Measured with achiral method D.

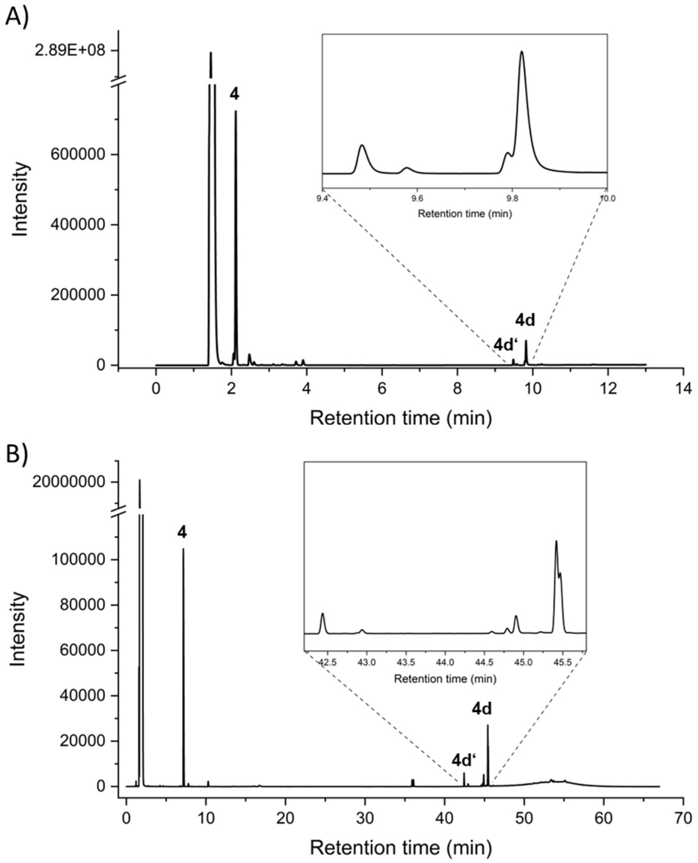

Figure S18: Achiral (A) and chiral (B) GC-chromatogram of the reaction using purified HheG3, methyl-epoxy-cyclohexane (**4**) as substrate and thiocyanate as nucleophile after 24 h. Measured with achiral and chiral method A.

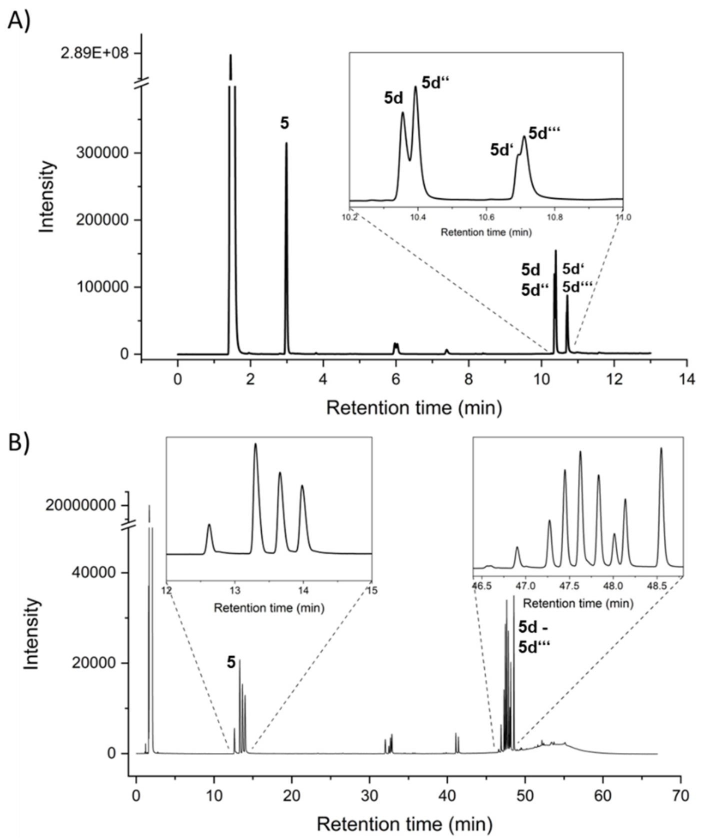

Figure S19: Achiral (A) and chiral (B) GC-chromatogram of the reaction using purified HheG3, epoxy-vinyl-cyclohexane (**5**) as substrate and thiocyanate as nucleophile after 24 h. Measured with achiral and chiral method A.

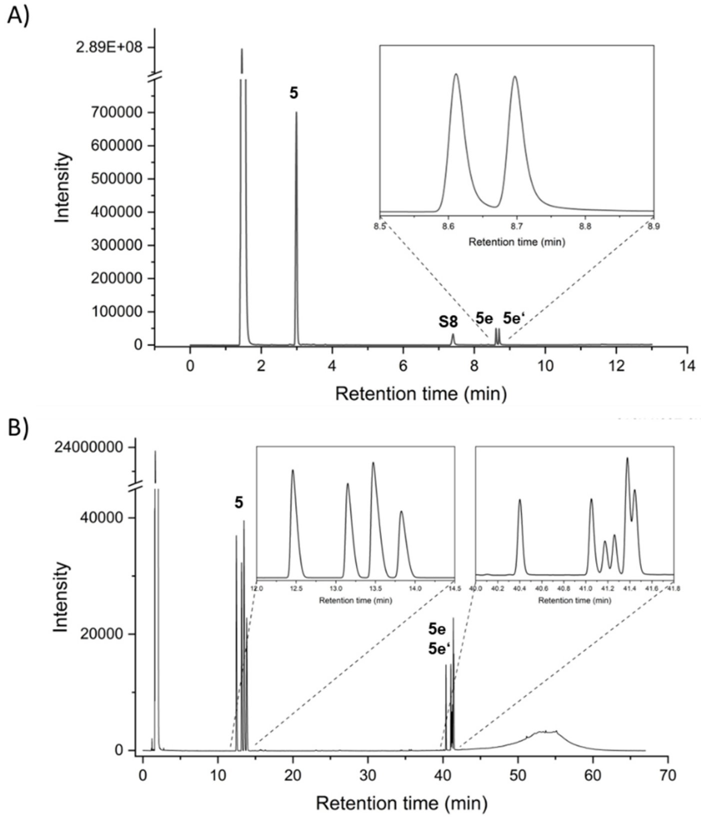

Figure S20: Achiral (A) and chiral (B) GC-chromatogram of the reaction using purified HheG3, epoxy-vinyl-cyclohexane (**5**) as substrate and formate as nucleophile after 24 h. Measured with achiral and chiral method A.

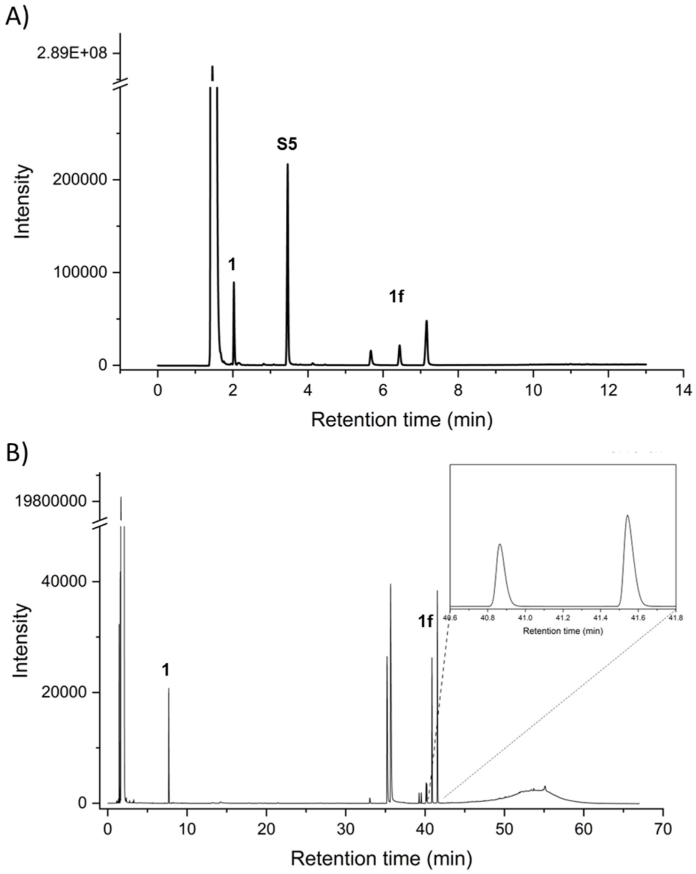

Figure S21: Achiral (A) and chiral (B) GC-chromatogram of the reaction using purified HheG3, cyclohexene oxide (**1**) as substrate and nitrite as nucleophile after 24 h. Measured with achiral and chiral method A.

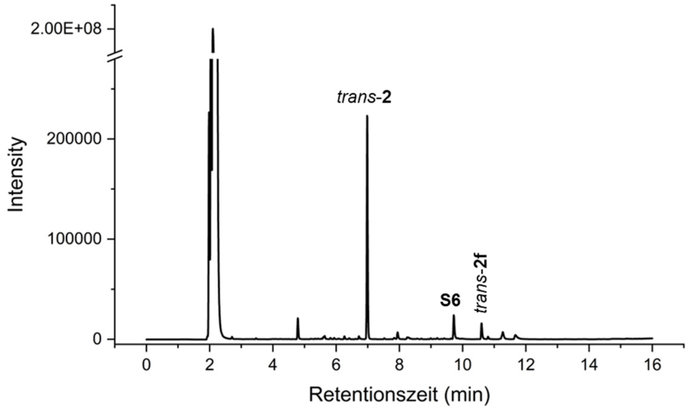

Figure S22: Achiral GC-chromatogram of the reaction using purified HheG3, (+)-*trans*-limonene oxide (**2**) as substrate and nitrite as nucleophile after 24 h. Measured with achiral method D.

Supplementary references

(1) Brand, A.; Allen, L.; Altman, M.; Hlava, M.; Scott, J. Beyond Authorship: Attribution, Contribution, Collaboration, and Credit. *Learn. Publ.* **2015**, *28* (2), 151–155. https://doi.org/doi:10.1087/20150211.

(2) Kaspar, F.; Crameri, F. Coloring Chemistry—How Mindful Color Choices Improve Chemical Communication. *Angew. Chemie Int. Ed.* **2022**, e202114910. https://doi.org/https://doi.org/10.1002/anie.202114910.

(3) Gasteiger, E.; Hoogland, C.; Gattiker, A.; Duvaud, S.; Wilkons, M. R.; Appel, R. D.; Bairoch, A. Protein Identification and Analysis Tools on the ExPASy Server. In *The Proteomics Protocols Handbook*; Walker, J., Ed.; Humana Press, 2005; pp 571–607.

(4) Mirdita, M.; Schütze, K.; Moriwaki, Y.; Heo, L.; Ovchinnikov, S.; Steinegger, M. ColabFold: Making Protein Folding Accessible to All. *Nat. Methods* **2022**, *19* (6), 679–682. https://doi.org/10.1038/s41592-022-01488-1.

(5) Zhang, Z.; Miller, W.; Schäffer, A. A.; Madden, T. L.; Lipman, D. J.; Koonin, E. V; Altschul, S. F. Protein Sequence Similarity Searches Using Patterns as Seeds. *Nucleic Acids Res.* **1998**, *26* (17), 3986–3990. https://doi.org/10.1093/nar/26.17.3986.

(6) Marcus, S.; Julia, K.; Elizabeth, W.; Rainer, W.; Anett, S.; E., P. R. Expanding the Halohydrin Dehalogenase Enzyme Family: Identification of Novel Enzymes by Database Mining. *Appl. Environ. Microbiol.* **2014**, *80* (23), 7303–7315. https://doi.org/10.1128/AEM.01985-14.

(7) Katoh, K.; Standley, D. M. MAFFT Multiple Sequence Alignment Software Version 7: Improvements in Performance and Usability. *Mol. Biol. Evol.* **2013**, *30* (4), 772–780. https://doi.org/10.1093/molbev/mst010.

(8) Kaspar, F.; Solarczek, J. Supplementary Material. **2022**. https://doi.org/10.5281/zenodo.6785962.

(9) Koopmeiners, J.; Diederich, C.; Solarczek, J.; Voß, H.; Mayer, J.; Blankenfeldt, W.; Schallmey, A. HheG, a Halohydrin Dehalogenase with Activity on Cyclic Epoxides. *ACS Catal.* **2017**, *7* (10), 6877–6886. https://doi.org/10.1021/acscatal.7b01854.

(10) Solarczek, J.; Klünemann, T.; Brandt, F.; Schrepfer, P.; Wolter, M.; Jacob, C. R.; Blankenfeldt, W.; Schallmey, A. Position 123 of Halohydrin Dehalogenase HheG Plays an Important Role in Stability, Activity, and Enantioselectivity. *Sci. Rep.* **2019**, *9* (1), 5106. https://doi.org/10.1038/s41598-019-41498-2.

(11) Schallmey, M.; Jekel, P.; Tang, L.; Majerić Elenkov, M.; Höffken, H. W.; Hauer, B.; Janssen, D. B. A Single Point Mutation Enhances Hydroxynitrile Synthesis by Halohydrin Dehalogenase. *Enzyme Microb. Technol.* **2015**, *70*, 50–57. https://doi.org/https://doi.org/10.1016/j.enzmictec.2014.12.009.

(12) Bergmann, J. G.; Sanik, J. Determination of Trace Amounts of Chlorine in Naphtha. *Anal. Chem.* **1957**, *29* (2), 241–243. https://doi.org/10.1021/ac60122a018.

(13) Eilert, L.; Schallmey, A.; Kaspar, F. UV-Spectroscopic Detection of (Pyro-)Phosphate with the PUB Module. *Anal. Chem.* **2022**, *94* (8), 3432–3435. https://doi.org/10.1021/acs.analchem.1c05356.

(14) Wright, T. A.; Stewart, J. M.; Page, R. C.; Konkolewicz, D. Extraction of Thermodynamic Parameters of Protein Unfolding Using Parallelized Differential Scanning Fluorimetry. *J. Phys. Chem. Lett.* **2017**, *8* (3), 553–558. https://doi.org/10.1021/acs.jpclett.6b02894.
